# Discovery and annotation of putative genes and novel RNA splice-isoforms in the human heart

**DOI:** 10.3389/fcvm.2026.1920242

**Published:** 2026-09-29

**Authors:** Kristina Santucci, Yuning Cheng, Yulan Gao, Konii Takenaka, Grace Lindner, Si-Mei Xu, Michael Janitz

**Affiliations:** School of Biotechnology and Biomolecular Sciences, University of New South Wales, Sydney, NSW, Australia

**Keywords:** alternative splicing, cardiac transcriptome, heart, long-read sequencing, novel isoform

## Abstract

The analysis of long-read RNA sequencing (RNA-seq) data can extend current annotations across the genomes, transcriptomes, and proteomes of even the most annotated species such as *Homo sapiens*. Long-read sequencing (LRS) technologies like Oxford Nanopore Technologies' (ONTs) nanopore sequencing can capture full-length transcripts, making the task of read-to-isoform assignment less challenging. This enables both the discovery of new RNA splice isoforms as well as a more accurate measure of their individual expression. RNA expression from unannotated regions of the genome can also lead to the discovery of putative (candidate novel) genes. Computational methods such as protein-coding potential assessment and ORF sequence prediction allow for the detection of new coding sequences (CDS), which can expand proteome annotations. This study seeks to apply these methods for the discovery of new RNA isoforms deriving from both annotated and putative genes in the human cardiac transcriptome. Recent LRS of prized Genotype Tissue Expression (GTEx) project human samples of the atrial appendage, left ventricle, and skeletal muscle were re-analysed with a new analytical pipeline that seeks to robustly discover these novel features. As a result, 54 intergenic and 112 antisense putative genes were detected. 491 novel RNA isoforms were discovered, 173 of which were transcribed from putative genes. 142 of the novel RNA isoforms were determined to have coding potential, and the remaining 349 determined to have limited or no coding potential with lengths greater than 200 nt, deeming them long non-coding RNAs (lncRNAs). Of the messenger RNA (mRNA) isoforms, 56 had low homology to known proteins, suggesting they may encode new protein isoforms or truncated proteins, and 24 had no detected homology to known proteins. Overall, this study has characterised new genes, RNA molecules, and protein isoforms, and has explored their potential function through various sequence and expression-based analyses, including the detection of conserved sequence and protein domains, cis-regulatory potential analysis, and weighted gene co-expression network analysis. Such analyses consistently demonstrated the potential for these discovered features to have functional roles in the human cardiac system. By re-analysing existing datasets, this study has revealed information that was not captured in previous analyses, resulting in more complete and accurate reference annotations of the human genome.

## 1 Introduction

Alternative splicing is a well characterised molecular mechanism that enables the production of multiple RNA transcripts from a single gene. It allows for a limited number of protein-coding genes in the human genome to produce a functionally diverse proteome (1). Alternative splicing also takes place for non-protein coding genes, expanding the repertoire of significant regulatory features such as lncRNAs, which play a major role in the modulation of gene expression and have a demonstrated influence on alternative splicing (2–4). Modulation of splice-isoform usage can be observed in developmental processes, cell state changes and a variety of diseases, with an estimated 15% of human hereditary diseases and cancers having a known association with alternative splicing (5–9). The human brain transcriptome expresses more than 76% of protein-coding genes (10–12) with abundantly characterised splice isoforms over numerous transcriptomic studies (13, 14). Human peripheral tissues also demonstrate pervasive alternative splicing. An extensive review was completed on the human cardiac transcriptome, which detailed the vast influence of several cardiac splicing factors that subsequently can affect the function of cardiomyocytes (15). Numerous cardiac pathologies, including cardiomyopathy and arrhythmia, have been associated with the alteration of alternative splicing of genes that encode functional proteins such as ion channels (15).

Long-read RNA-seq provides and opportunity to improve genome, transcriptome, and proteome annotations (16, 17). LRS technologies such as ONT's nanopore sequencing do not require RNA fragmentation prior to sequencing. Without this, RNA molecules can be sequenced natively producing reads lengths of 10–100 kb, and over 4 Mb. This property of the technology can capture the full length of RNA transcripts, reducing the computational challenge of reconstructing and quantifying RNA isoforms from individual reads (17, 18). As a result, the discovery of new RNA splice isoforms and putative genes across several species has been achieved (18, 19–24). Despite this, there are significant challenges in this field of discovery driven research, primarily due to the computational constraints of base-calling software and novel transcript modelling (25–27). The Long-read RNA-seq Genome Annotation Assessment Project (LRGASP) recently identified these challenges, highlighting the variability in the set of unannotated transcript models produced when utilising different transcriptome assemblers (28). This was also demonstrated in a recent study, where the overlap of the sets of novel transcripts produced by IsoQuant, Bambu, and FLAIR is minimal, indicating the choice of transcriptome assembly tool for long-read RNA-seq data is a major source of bias for the detection of unannotated features (14). Researchers have attempted to reduce the inclusion of false-positive novel isoforms in transcriptomic studies by employing machine-learning and rule-based algorithms to remove spurious isoforms from the assembly (29, 30). These unannotated RNA isoforms can be further characterised using the widely adopted SQANTI conventions, including terms such as novel in catalogue (NIC) where a new combination of known splice sites and or junctions are featured in transcripts, novel not in catalogue (NNC), where new features such as splice sites or exons, genic isoforms that cover both exonic and intronic region of annotated gene, and fusions RNAs (31). For RNA isoforms transcribed from unannotated genes, they can also be classed using the SQANTI conventions as intergenic, for those situated between two annotated genes, antisense, where the transcript is in the antisense orientation to an annotated gene, or genic introns, which are completely contained within the intron of an annotated gene (typically intronic lncRNAs) (32).

This study aims to identify and characterise the potential functional roles and tissue-specific expression patterns of new genes, RNA isoforms, and protein isoforms in two of the three types of muscle tissues: cardiac and skeletal muscle. This characterisation will focus on cardiac tissues derived from the atrial appendage and left ventricle of the human heart, with comparisons of these tissues' transcriptomes made against one another and against skeletal muscle. Here, we aim to identify portions of the genome and transcriptome that are unique to such cardiac tissues, using skeletal muscle as a comparator, as cardiac and skeletal muscle cells share features in structure, function, and physiology. This study robustly detected and characterised novel features by utilising a comprehensive and stringent analytical pipeline that aims to minimise the inclusion of sequencing artefacts and reduce bias introduced by only using individual computational algorithms. This was achieved by incorporating both rules-based and ML filters at various stages of the analytical pipeline, as well as adopting the consensus set of multiple programs that have the same functionality at both the transcript construction and characterisation steps. Additionally, separate focused analyses on both lincRNA genes and protein-coding mRNAs were carried out. Here, the cis-regulatory potential of lincRNA genes on their most proximal protein coding gene was assesesed. For mRNAs, the proteins' tertiary structure will be predicted from the mRNA ORF, and its potential functions will be explored. This study seeks to highlight the importance of using robust computational methods, as the inclusion of false-positive novel features in genome annotation from less stringent methods can generate abundant noise that crowds genuine novel and biologically relevant aspects of the transcriptome.

## 2 Methodology

The overall analytical pipeline used in this study is summarised in Supplementary Figure S1. This is broadly structured into three main steps; data pre-processing and alignment, transcriptome assembly and novel feature discovery, and finally, the characterisation of such novel features including genes, transcripts, and protein isoforms.

### 2.1 Data retrieval

#### 2.1.1 GTEx v9 data

Nanopore bulk cDNA sequencing data was obtained from the National Centre for Biotechnology Information (NCBI) database of Genotypes and Phenotypes (dbGaP) using the accession number phs000424.v9 (33–35). This sequencing data was produced as part of the GTEx v9 project (36). This cDNA was derived from healthy human tissues, including nine biological replicates of the atrial appendage, seven biological replicates of the left ventricle, and nine biological replicates of skeletal muscle.

#### 2.1.2 GTEx v8 data

Illumina bulk cDNA sequencing data was accessed from the GTEx v8 project (35). This included three biological replicates each of healthy human tissues, including the atrial appendage, left ventricle, and skeletal muscle. This data was used for the visualisation and comparison of raw reads to that of LRS samples in Integrative Genomics Viewer.

#### 2.1.3 CAGE peak data

Cap Analysis Gene Expression (CAGE) peak data was accessed from the refTSS4 (human 4.1) dataset. This is a reference database for transcription start sites (TSSs), a curation of various public resources as described by (37).

#### 2.1.4 3′-end sequencing data

*Homo sapiens* (GRCh38.96) 3′-end sequencing data was accessed from the PolyASite atlas (v2.0). This 3′-end sequencing data atlas is comprised of 221 contributing samples, utilising the following sequencing protocols; 3′-Seq (Mayr), 3′READS, DRS, QuantSeq_REV, SAPAS, PAPERCLIP, PolyA-seq, PAS-Seq, A-seq, 3P-Seq.

### 2.2 Raw quality check and quality control

Summaries of the dataset's quality were produced using NanoComp v1.23.1 (38). After quality assessment, Nanofilt (39) 2.8.0 was used to filter out reads with a Phred quality q-score below 10. Another quality report was produced post-read filtering using NanoComp v1.23.1.

### 2.3 Sequencing data alignment

All quality-controlled samples were subjected to a splice junction aware alignment with Minimap2 and 2passtools (30) to the Genome Reference Consortium Human Build 38 patch 14 (GRCh38.p14) genome (GENCODE v49). The purpose of this two-pass alignment is to improve downstream transcript assembly at difficult and error prone regions, including at splice junctions and small exons. Older Nanopore sequencing kits, such as those used to generate the project data, are notorious for a drop in accuracy at splice sites, thus it is critical to filter these low-accuracy sites before transcriptome assembly with the goal of discovering new genes and isoforms, as they may mistakenly feature new splice sites. This reduces the chance of including false positive putative genes and isoforms that had arisen from sequencing artifacts.

Sequencing reads were first aligned with Minimap2 v2.24 (40) using the tags *-a –cs* *=* *long -k14 -x splice.* The SJs from the first alignment were analysed and scored with the machine learning algorithm 2passtools v0.3.1 using the tag *–score* (30). 2passtools ranks the SJs using a logistic regression model that extracts sequencing information and metrics such as the number of supporting reads and splice junction sequence motifs. The low scoring SJs were removed from the annotated junction.bed file using the 2passtools *filter* module with all default filter thresholds (30). The filtered reads were then aligned again (second-pass alignment) with Minimap2 using the tags *-a –cs* *=* *long -k14 -x splice –junc-bed,* with the addition of supplying the filtered junction.bed files. The final aligned.sam files were converted into bam format using Samtools v1.15.1 with tags *-Sb*.

### 2.4 Transcript assembly

Following read alignment, transcripts were assembled and quantified with two different methods; Bambu v3.12.1 (41) and IsoQuant v3.6.3 (42). Both assembly methods were alignment based, supplying the same annotation files used for read alignment (GENCODE version 49). Transcriptome assemblies with IsoQuant were performed using the default nanopore mode (–data_type nanopore). The aligned.bam files were supplied to IsoQuant using the parameter *–yaml,* so expression values could be calculated and normalised across all replicates. This also increases the precision of the novel isoform structure realisation and quantification.

Assembly with Bambu was also performed simultaneously across all sample replicates, using a novel discovery rate (NDR) threshold of 0.083. Using the default Bambu NDR settings, the NDR threshold of 0.083 was calculated based on the level of annotation completeness of the input data. This is calculated by comparing the data to a model trained on human reference annotations (Hg38) (41). The NDR is the proportion of novel transcripts among the set of all assembled (novel and annotated) transcripts with an equal or higher transcript score. The transcript score is defined by Chen et al. (41) as the validity likelihood of a transcript in at least one sample. Principally, the NDR threshold is an upper limit on the false discovery rate, such that an NDR of 0.083 suggests that approximately 91.7% of transcripts with an equal or higher transcript score are annotated, and 8.3% are novel (41). For well-annotated organisms, a lower NDR threshold (NDR 0.05–0.1) prevents the overestimation of new isoforms, thus increasing the precision of detection. Bambu was run in an R v4.51 environment.

### 2.5 PCA and hierarchical clustering

The Bambu transcriptome assembly underwent two-dimensional principal component (PCA) analysis. The standard deviation (SD) of normalised expression values (CPM) was calculated for each transcript, as well as a median SD of all CPM values. Only transcripts with variable expressions, defined as those with SD > median SD, were used for PCA analysis. For hierarchical clustering, Spearman's Correlation Coefficient (SCC) was calculated using log-base 2 transformed CPM values [log2(CPM + 1)]. The PCA and hierarchical clustering analysis plots were generated with Bambu in an R v4.51 environment.

### 2.6 Transcript model characterisation and quality control

Novel RNA isoforms from Bambu and IsoQuant transcriptome assemblies were characterised and quality controlled using SQANTI-qc v5.5 (61) This quality control module classifies annotated and novel transcripts based on pre-defined structural categories and subcategories (31, 32). The classification of novel RNA isoforms transcribed from annotated genes is based on structural comparisons, such as exon-skipping events and 3′ and/or 5′ fragments. This would class the transcripts as NIC (new combination of known splice sites/junction), NNC (new splice sites), fusions, or genic isoforms (cover exonic and intronic region of annotated gene). For RNA isoforms that map to an unannotated locus, the classification is based on the annotated genes within proximity of that locus, defining them as intergenic (between two annotated genes), antisense (antisense orientation to known gene), or genic introns which are completely contained within the intron of an annotated gene. CDS detection and ORF prediction were also performed using the TransDecoder2 algorithm (43), defining transcripts as non-coding or having protein-coding potential.

For downstream filtering, CAGE peak data from refTSS4 (human 4.1) and the Homo sapiens PolyASite (v2.0) (GRCh38.96) 3′-end sequencing data atlas (44) were supplied to SQANTI3-qc to evaluate the 5′ and 3′ ends of transcript models. The parameter *–ratio_TSS_metric* was set to *mean*, calculating the mean coverage 100 bp upstream and downstream the reported TSS. A list of common polyA motifs available from the SQANTI3 program was also supplied to the quality control module. This included the following common human polyA motifs;

**Table:** 

aataaa	cataaa	aataga	aatgaa
attaaa	gataaa	aaaaag	tttaaa
agtaaa	aatata	actaaa	aaaaca
tataaa	aataca	aagaaa	ggggct

The quality-controlled and characterised assemblies were subsequently filtered with the SQANTI-filter module. Here, a custom rules-based filter was designed and supplied to the module with *-j* argument. This rule-based filter identifies and removes transcript models suspected to be sequencing artifacts, leaving only models suspected to be true positive isoforms. The filter is structured in four rules, those pertaining to full-splice matches (FSM) (i.e., reference transcripts), NIC transcripts, NNC transcripts, and the remaining transcript models, such as incomplete splice matches (ISM). For FSMs, they were filtered if they were suspected to be the result of an intra-priming event, or the result of a reverse transcriptase template switching (RT-switching) event. Transcripts were flagged as a potential intra-priming event if there was a 60% adenosine stretch after the transcription termination site (TTS), defined as 12 or more adenines present in the 20 bp genomic window downstream of the TTS (32, 45). For novel isoforms under the NIC and NNC structural categories, transcripts were filtered out if they were mono-exonic, suspected to be the result of intra-priming event, or the result of a RT-switching event. For novel transcript models that contained all canonical splice junctions, no additional filtering was applied. For those with non-canonical splice junctions, the transcript models also had to satisfy the condition that the absolute distance to the closest annotated TSS and TTS was 50 bp or less. ISMs were excluded as a novel class as they represent alternative transcripts shorter at the 3′ and/or 5′ end, which may be caused by RNA degradation. For the remaining transcripts, the transcripts were filtered out if they were mono-exonic, suspected to be the result of intra-priming event, the result of a RT-switching, or contained non-canonical splice junctions.

### 2.7 Transcriptome assembly merging for putative gene and RNA discovery

After filtering the IsoQuant and Bambu transcriptome assemblies, a set of novel isoforms that were structurally equivalent across both assembly tools was curated. This further reduces the inclusion of false positives as the set of novel isoforms and genes is biased to the assembly method chosen. GffCompare v0.11.2 (46) was used for the curation by supplying the transcriptome assemblies from Bambu and IsoQuant in Gene Transfer Format (GTF) for transcript matching. A compiled set of non-redundant transcripts was generated in the tracking (.tracking) file, where each transcript model is identified as present or absent in each supplied assembly. GffCompare considers transcripts to be structurally equivalent between two samples (or in this case assemblies) if their introns are identical. Transcripts can differ in their length but only if this is due to the length of the first and or last exon, as this is variable across both biological samples and assembly methods. Using the AWK programming language, the set of novel transcripts present in (and thus structurally equivalent across) both assemblies were extracted. This non-redundant list was further reduced by removing the artifactual transcript models identified by the rules filter. Novel isoforms were renamed for convenience, indicating whether they have protein-coding potential.

### 2.8 Visualisation of isoforms

Putative gene and RNA isoform visualisations were produced with Bambu 3.12.1 (41) in an R v.4.51 environment. The exonic-intronic structure of putative genes and encoded RNA/s were visualised at their genomic location. The structure of novel isoforms from annotated genes were visualised against annotated isoforms derived from the same locus. For all RNAs (annotated RNAs, putative gene derived RNAs, and annotated gene-derived RNAs) their base-2 logarithmic transformed expression values [log2(CPM + 1)] were visualised in a heatmap for all sample replicates. This was also produced with Bambu 3.12.1 (41) in an R v.4.51 environment.

### 2.9 Differential gene expression analysis

Differential gene expression (DGE) analysis was performed under R v.4.51 on the Bambu transcriptome assemblies with DESeq2 v.1.50.2 (47) (based on the negative binomial distribution). For this analysis, individual transcript expression counts were first converted to gene expression counts with the Bambu *transcriptToGeneExpression* function. The raw gene count data was supplied to DESeq2 were utilised for DGE analysis. There were three DGE analyses with contrasts as follows; left ventricle against skeletal muscle, atrial appendage against skeletal muscle, and left ventricle against atrial appendage. Statistical significance was calculated using the Wald-test method (*p*-value < 0.05) following the DESeq2 default multiple testing correction, which is an interpretation of the Benjamini-Hochberg procedure [adjusted *p*-value (*p.adj*) < 0.1]. For downstream visualisations, the shrunken log2 fold changes (LFC) values were calculated with the DESeq2 *lfcShrink* function using the ashr v2.2-63 (48) package. MA plots were generated with BiocGenerics v0.56.0 (49), and volcano plots were generated with EnhancedVolcano v1.28.2 (50). Karyotype plots were generated with karyoploteR v1.36.0 (51) using the BSgenome.Hsapiens.UCSC.hg38 v.1.78.0 genome package and GenomicRanges v.1.62.1 (52) package *makeGRangesFromDataFrame* function.

### 2.10 Cis-regulatory potential of novel lincRNA genes

A Spearman's correlation analysis was performed on the gene expression of novel lincRNA genes and their closest annotated protein-coding gene. The distance to and identification of the closest annotated protein-coding genes was determined with Genomic Ranges *distanceToNearest* function in an R v.4.51 environment (52). Correlations were tested with Spearman's *rho* and *p*-values were adjusted with the Benjamini and Hochberg (BH) method, also known as the false discovery rate (FDR) (53). The signed log_10_(|distance|) and actual distance of each novel lincRNA from their nearest protein-coding gene was plotted with ggplot2 v4.0.3 and ggrepel v0.9.8 in an R v.4.51 environment (54).

### 2.11 Weighted gene co-expression network analysis

To explore the potential functional roles of putative genes, weighted gene co-expression network analysis (WGCNA) was performed on all annotated and putative genes across the 25 samples (55). This method allows for the transcriptomic investigation of relationships between all genes in a system. These relationships can be used to form clusters or small networks of genes known as modules, that bear a similar expression profile across the samples. This expression profile is known as an eigengene. WGCNA can also incorporate external characteristics of the dataset, including both quantitative and qualitative data. As a result, the correlation between eigengenes and such traits can be determined. Here we aim to identify clusters of genes whose expression pattern over the 25 samples is significantly correlated with tissue type. Further, by determining the functional roles of hub genes in such networks by gene ontology and Kyoto Encyclopedia of Genes and Genomes (KEGG) pathway enrichment analysis (56, 57), we aim to infer the potential functional roles of putative genes within these clusters via guilt-by-association.

#### 2.11.1 Gene and sample filtering

Raw gene count values were normalised using a variance stabilizing transformation DESeq2 v.1.50.2. The variance-stabilised dataframe was transposed and filtered for application to WGCNA using the *goodSamplesGenes* function from the WGCNA v.1.74 package (55). This function identifies missing entries, entries with weights below a threshold, and zero-variance genes. Such genes (*n* = 31,838) were subsequently filtered out leaving those that are appropriate for WGCNA (*n* = 47,310). Hierarchical clustering using the “average” method was performed on the 25 samples with *hclust* (flashClust v1.1-4) to identify potential sample outliers. All 25 samples were not deemed outliers and were retained for the analysis.

#### 2.11.2 Weighted adjacency matrix construction

WGCNA ultilises a soft-thresholding approach to determine correlation as opposed to hard-thresholding, which defines a correlation cutoff. Hard-thresholding considers genes with a correlation greater than the threshold as connected. Soft-thresholding raises the correlation values to a power β. This type of thresholding will weaken small gene correlations and emphasise stronger ones, whilst not excluding potentially meaningful connections that would otherwise be excluded from a hard-threshold. The power β to calculate the adjacency matrix for a signed network was selected using the WGCNA *pickSoftThreshold* function. The first power β to exceed a scale-free topology fit index (R^2^) of 0.80 was selected. On this basis, a *β* = 16 was chosen (R^2^ = 0.93, mean connectivity = 157.5). The weighted adjacency matrix was constructed using the WGCNA *adjacency* function. Here, the pairwise correlations between all genes was calculated using Pearsons correlation. The Pearson correlation coefficients (PCCs) were then used to calculate the strength of the connection between each pair of genes. This was achieved by converting the correlation into signed similarity measures raised to a power of 16, such that adjacency = PCC^β^, where the sign signifies positive or negative correlation.

#### 2.11.3 Topological overlap matrix construction and dissimilarity calculation for gene clustering and module identification

The adjacency matrix is used to construct a Topological Overlap Matrix (TOM) using the WGCNA *TOMsimilarity* function. This not only identifies whether genes in each pair are connected to each other, but also how similar their connections to all other genes are. The resulting TOM was converted into a dissimilarity matrix by subtracting each TOM value from 1. This dissimilarity matrix was used for average-linkage hierarchical clustering with the flashClust v1.1-4 *hclust* function to construct a gene dendrogram for module identification. Dynamic tree cutting was then applied to identify modules with the following parameters; *deepSplit = 2, pamRespectsDendro = FALSE, minClusterSize = 30* (dynamicTreeCut v.1.63-1 *cutreeDynamic* function). The module numbers were converted to colours for visualisation against the gene dendrogram using the WGCNA *labels2colors* function. Each resulting module contains a set of genes whose expression profiles across the 25 samples are highly correlated. The grey module (Module 0) is the exclusion to this rule, containing only genes whose expression profile was not correlated with any module (*n* = 793).

#### 2.11.4 Calculating module eigengenes and merging of highly correlated modules

A module eigengene is a standardised gene expression profile of a module, such that it represents the dominant gene expression pattern across all samples. Module eigengenes were determined with the WGGNA *moduleEigengene* function. To merge modules with similar eigengenes, Pearson's correlation was applied using the WGCNA *cor* function with the parameter *use = complete.* Measures of dissimilarity between eigengenes were then calculated by subtracting each correlation value from 1. Modules with correlation values greater than 75% (dissimilarity < 0.25) were merged using the WGCNA *mergeCloseModules* function (Supplementary Figure S2). The dendrogram with the coloured modules, pre and post merging, was visualized using the WGCNA *plotDendroAndColors* function (Supplementary Figure S3).

#### 2.11.5 Evaluation of module-tissue, gene-module, and gene-tissue associations

To assess whether any identified modules were significantly correlated with one of the tissues, a binary dataframe was constructed on the 25 samples based on their tissue of origin. Correlation was calculated using Pearson's correlation between module eigengenes and tissues, and the associated significance values were determined using a student asymptotic *p*-value. The correlations and *p*-values were plotted with the WGCNA *labeledHeatmap* function. The correation between eigengenes and tissues were plotted with WGCNA *plotEigengeneNetworks* function (Supplementary Figure S4).

To further analyse the gene networks within modules significantly correlated with the atrial appendage or left ventricle, intramodular gene connectivity was explored. This was achieved by calculating gene module membership which measures how strongly the expression profile of each gene across all samples correlates with a module eigengene. This was determined using Pearson's correlation, and significance was measured using student asymptotic *p*-values. Then, three gene-trait significance values were determined between all genes and each of the three tissue types. This was achieved using the same statistical tests for correlation and associated *p*-values described for module membership. These values indicate the strength of association between a gene and a tissue. Scatterplots were produced with the WGCNA *verboseScatterplot* function for modules of interest.

#### 2.11.6 Gene ontology and KEGG pathway enrichment analysis of hubgenes

Next, intramodular connectivity was measured to identify hub gene in modules significantly correlated with individual heart tissues. For the modules of interest, the intramodular connectivity was calculated using a signed adjacency matrix with a soft-power of 16. Connection weights were summed for each gene in the adjacency matrix (−1 to not count self-connection). The top 10% of the most connected genes in the module of interest were identified as hub genes. Such genes were subjected to GO enrichment using the clusterProfiler v.4.18.4 *enrichGO* function (56, 58). The enrichment was performed on biological process ontology with a *p*-value and q-value cutoff of 0.05. The *p*-value adjustment method was Benjamini-Hochberg correction. For KEGG pathway enrichment, gene symbols were first converted to Entrez ID's using the clusterprofiler *bitr* function. The enrichment was then performed using the clusterProfiler *enrichKEGG* function with the same *p*-value and q-value paramteres as the GO enrichment analysis (57, 59). Concept networks were constructed with the clusterprofiler *cnetplot* function.

### 2.12 Protein-coding RNA sequence characterisation

The coding potential of each transcript was calculated using the TransDecoder2 algorithm (43) as part of the SQANTI-qc module. ORF sequences were also detected for transcripts predicted to be protein-coding. Novel RNAs with predicted ORFs underwent further characterisation and analysis, described in the following sections. The goal of these analyses was to identify novel proteins encoded by novel mRNAs from putative genes, as well as novel protein isoforms, encoded by novel mRNAs from annotated genes.

#### 2.12.1 Protein sequence homology

To start, the amino acid sequence of the predicted ORF of novel mRNAs were compared to known protein sequences using the protein-protein Basic Local Alignment Search Tool (blastp) (60) to identify whether such ORFs were already identified and characterised by other studies. The blastp search against the ClusteredNR (nr_cluster_seq) database for *Homo sapiens* (taxid:9606) was performed with 250 maximum target sequences and the remaining search parameters as default. A second blastp search was performed for all organisms against the ClusteredNR database, increasing the maximum target sequences to 500 to identify protein matches for those with no human matches.

#### 2.12.2 Protein domain and conserved domains search

Novel proteins and novel protein isoforms of interest were identified as those with low or no homology to known proteins. This was assessed manually for each transcript, taking into account the percent identity scores and sequence coverage. Manual evaluation of homology was conducted in this manner as the utilisation of an arbitrary percent identity cutoff and or sequence coverage cutoff either excluded genuine novel protein isoforms or included significant matches upon further inspection. For example, a novel transcript might match a known transcript for only 70% of the entire sequence, but has a 100% sequence match score for that portion (such that it may feature an alternative exon). ORF sequences of such proteins were uploaded to InterProScan to identify known protein domains from the InterProScan database of protein signatures (61). Further sequence characterisation was performed with NCBI Conserved Domain Search to identify conserved regions and domains present in the NCBI's Conserved Domain Database (CDD) (62).

#### 2.12.3 Sequenced-based topology prediction of alpha-helical transmembrane proteins

ORF sequences of interest were also analysed with TOPCONS v.2.0 (63) to identify transmembrane domains. If the provided ORFs (amino acid sequences) are from a putative alpha-helical transmembrane protein, the topology is predicted. TOPCONS topology prediction is a Hidden Markov Model, which analyses the predictions of five algorithms to provide a consensus. The topology prediction provides the estimated location of each amino acid (AA) as either intracellular, extracellular, or embedded within the membrane. The orientation is also provided for transmembrane segments, such that they are moving “out” or “in” (into) the cell. TOPCONS also predicts free energy (ΔG_app_), also known as the membrane insertion energy (ΔG) change (measured in kcal/mol), which is a measure of solubility such that low values suggest an energetically favoured membrane insertion. It is also determined if the ORF sequences have homology to a known 3D structure, and the topology of the homologous portions are shown.

#### 2.12.4 Protein structural prediction and assessment

The ORF sequences were uploaded to the AlphaFold3 (64) server for *ab initio* protein structural prediction. The SWISS-MODEL Structure assessment tool was utilised for further analysis of the AlphaFold3 protein prediction models. The tool integrates several algorithms for sequence annotation and model strength assessment.

#### 2.12.5 Structure-based topology prediction of alpha-helical transmembrane proteins

The QMEANBrane assessment (65) on the SWISS-MODEL server (66) was also performed on structures with TOPCONS identified transmembrane regions. This allows for the confirmation of alpha-helical transmembrane structure based on an implicit solvation model, as well as identifying which portions of the structure are soluble and what region is predicted to be embedded in the transmembrane. In comparison to the sequence-based prediction by TOPCONS, QMEANBrane makes the assessment by also taking the protein structure into account. The output shows whether the entire protein is likely to be in membrane environment or not. The overall insertion energy was provided and compared against that of known transmembrane proteins and non-transmembrane proteins.

### 2.13 Short-read RNA-seq validation of splice junctions

An independent set of short-read RNA-seq samples of the same GTEx v8 tissues were visualised alongside long-read RNA-seq replicates in Integrative Genomics Viewer (IGV) (67) v.2.16.2. As long reads produced from early Nanopore sequencing kits typically fall short in accuracy at splice junctions, this set of short-read samples was not only used to compare the ability of longer-read sequencing technologies to better capture full-length isoforms, but to also potentially validate new splice sites and junctions.

## 3 Results

### 3.1 Principal component analysis and hierarchical clustering

To provide an overview of how the transcriptomic profiles of the three samples compared against one another, a two-dimensional principal component analysis (PCA) and hierarchical clustering analysis was performed. As seen in Figure 1, the samples form distinct clusters based on the tissue of origin. The first principal component accounts for 24.2% of the total variation, whilst the second accounts for 8.9%. In both the dendrogram and heatmap of Supplementary Figure S5, it is observed that the two cardiac tissues have more similar RNA expression profiles than the skeletal muscle group, which is expected considering that both tissues are comprised of cardiac muscle cells.

**Figure 1:**
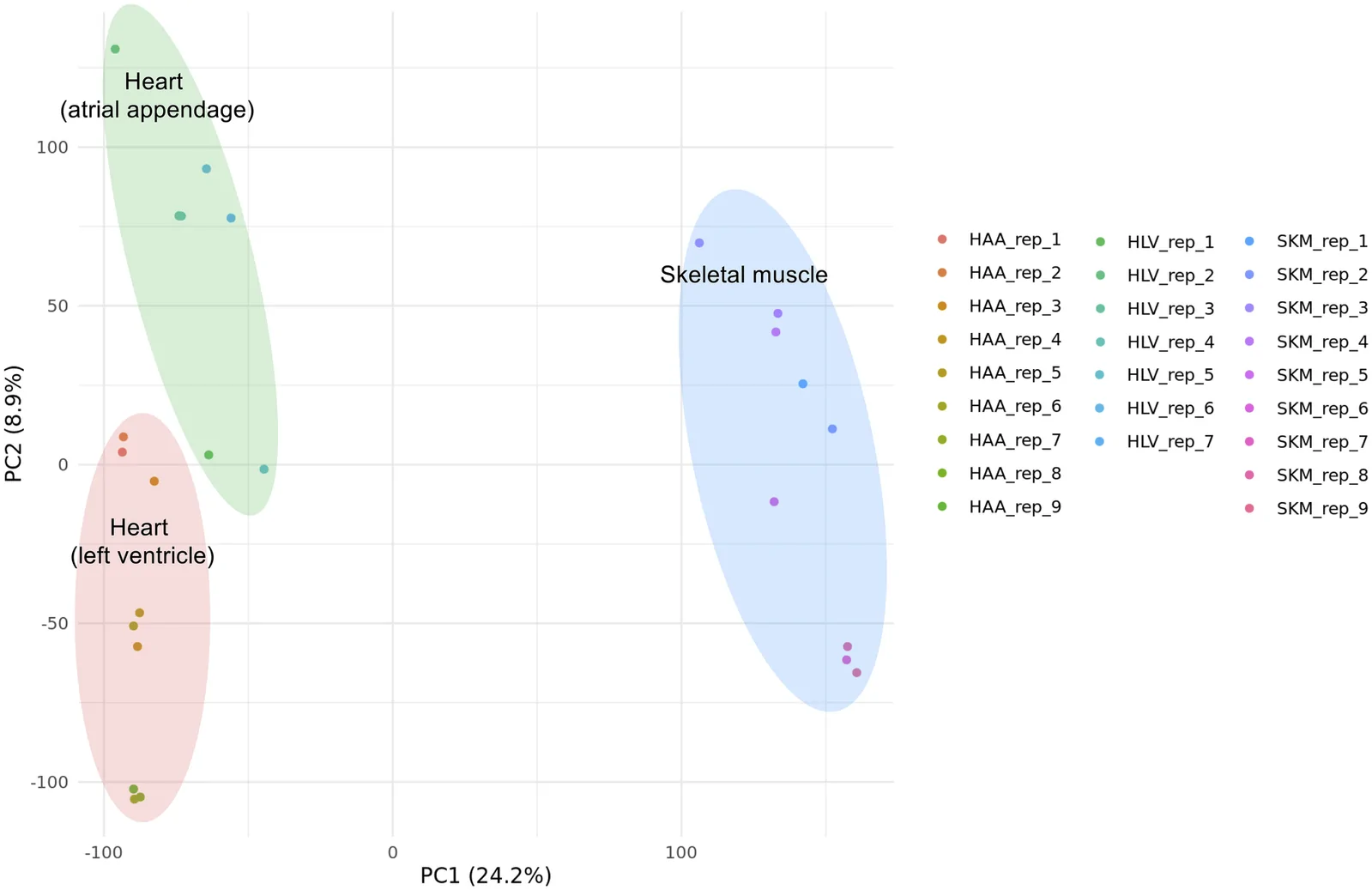
Principal component analysis of the atrial appendage, left ventricle, and skeletal muscle transcriptomes. Transcriptome assemblies of atrial appendage (*n* = 9), left ventricle (*n* = 7), and skeletal muscle (*n* = 9) were subjected to a two-dimensional PCA analysis. Transcripts were assembled with Bambu 3.6.0 (28) in an R (v4.40) environment and selected for PCA if the standard deviation (SD) of expression across samples was greater than the median SD. Normalised transcript expression values (CPM values) were log-base-2 transformed [log2(CPM + 1)]. The percentage of total variation by the first principal component (PC1) is shown on the *x*-axis, and second (PC2) on the *y*-axis. Individual replicates (dots) were highlighted according to their tissue of origin, with the atrial appendage in red, left ventricle in green, and skeletal muscle in blue. The analysis and plot were generated with Bambu 3.6.0 (28) in an R (v4.40) environment.

### 3.2 Transcriptome assembly, filtering, and characterisation for the identification of putative genes and novel RNA isoforms

Novel transcript models assembled by Bambu and IsoQuant were compared against one another, identifying those that are structurally equivalent across both methods. This resulted in a curated set of 583 novel isoforms, which was further filtered to reduce the downstream analysis of false positives. This filtering comprised of both the subjection to a rules-based SQANTI-filter (see methods) and manual inspection, resulting in the removal of 45 transcripts. Finally, 47 transcripts already discovered in the original study by Glinos et al. (36), who employed Flair for transcriptome assembly, were not further analysed.

As a result, 491 novel transcriptional isoforms remained in the final analysis set for downstream characterisation. Out of these novel isoforms, 166 are transcribed from putative genes, 112 of which are antisense to annotated genes. 142 out of the 491 novel RNA isoforms were determined to have coding potential, and the remaining 349 determined to have limited or no coding potential with lengths greater than 200 nt, deeming them lncRNAs. In terms of sub-category, There were 110 RNAs that featured new combination of known splice sites/junctions (NIC), 178 isoforms that featured new splice sites, 24 fusions RNAs, six genic isoforms (cover exonic and intronic region of an annotated gene), 39 intergenic (between two annotated genes), 119 antisense (antisense orientation to known gene), and 15 genic introns, which are completely contained within the intron of an annotated gene (generally intronic lncRNAs). The associated structural categories are outlined in Table 1 and presented in detail in Supplementary Table S1.

**Table 1:** Structural category of novel RNA isoforms.

Structural Category	Novel RNA (no.)
Antisense	119
Fusion	24
Genic	6
Genic intron	15
Intergenic	39
Novel in catalogue	110
Novel not in catalogue	178

The expression of these novel isoforms was also determined across the three tissues. Notably, most are expressed in all three tissues (*n* = 283), whilst 117 were expressed in only two of three (left ventricle and atrial appendage, *n* = 63; atrial appendage and skeletal muscle, *n* = 42; left ventricle and skeletal muscle, *n* = 12). Additionally, 91 isoforms were only expressed in one tissue (atrial appendage, *n* = 22; left ventricle, *n* = 0, skeletal muscle, *n* = 69).

### 3.3 Significantly differentially expressed putative genes

To identify significantly DEGs across the three tissues, three DGE analyses were performed for pair-wise comparisons. Comparing the left ventricle to skeletal muscle, 3,946 genes were significantly (*p.adj* < 0.1) upregulated (LFC > 1) out of 47,304 genes with a total read count greater than zero, accounting for ∼8.3% of expressed genes. Out of these genes, 12 were confirmed downstream as putative genes (Table 2). Following the same account, 2,732 (∼5.8%) genes were significantly downregulated (LFC < 1) with 34 of these being confirmed novel. Similarly, there were 5,305 (∼11.2%) significantly upregulated genes (*n* = 32; novel) in the atrial appendage in comparison to the skeletal muscle sample and 3,069 (∼6.5%) significantly downregulated genes (*n* = 44; novel). When comparing the left ventricle to the atrial appendage, there were 585 (∼1.2%) significantly upregulated (*n* = 3; novel) and 1,082 (∼2.3%) significantly downregulated (*n* = 4; novel) genes.

**Table 2:** Summary of differential gene expression analysis results for putative genes across three contrasts.

Comparison	HLV vs. SKM	HLV vs. HAA	HAA vs. SKM
Total significant*	46	7	76
Upregulated	12	3	32
Downregulated	34	4	44

HAA, heart atrial appendage; HLV, heart left ventricle; SKM, skeletal muscle.

* *p.adj* < 0.1, |LFC| > 1.

DGE results are summarised across various chromosome karyotype plots, that show the results for both annotated and putative genes. Significantly DEG are plotted in respect to their chromosomal location on the *x*-axis, and relative fold-change (log2FC) on the *y*-axis. The size of each point corresponds to the gene's expression level. For each pair-wise comparison, the top three significantly upregulated and downregulated putative genes are labelled. Chromosomes containing such genes of interest were represented for the three contrasts; left ventricle against skeletal muscle (Figure 2), atrial appendage against skeletal muscle (Figure 3), and left ventricle against atrial appendage (Supplementary Figure S6). The genomic location, LFC, and *pad.j* values of significantly differentially expressed putative genes are summarised in (Table 3). The novel antisense gene NovelGene_ENSG00000143603.20_AS is located on the sense strand of chr 1:154,707,537–154,708,570 (Figure 4A). The gene is significantly upregulated in the atrial appendage in comparison to skeletal muscle (*p.adj* = 7.03 × 10^−2^; LFC = 4.18). The gene was detected to encode one lncRNA isoform NoncodingTrx222, which is 763 nt long and comprised of two exons. The gene and lncRNA are antisense to the protein-coding gene Potassium Calcium-Activated Channel Subfamily N Member 3 (*KCNN3)*.

**Figure 2:**
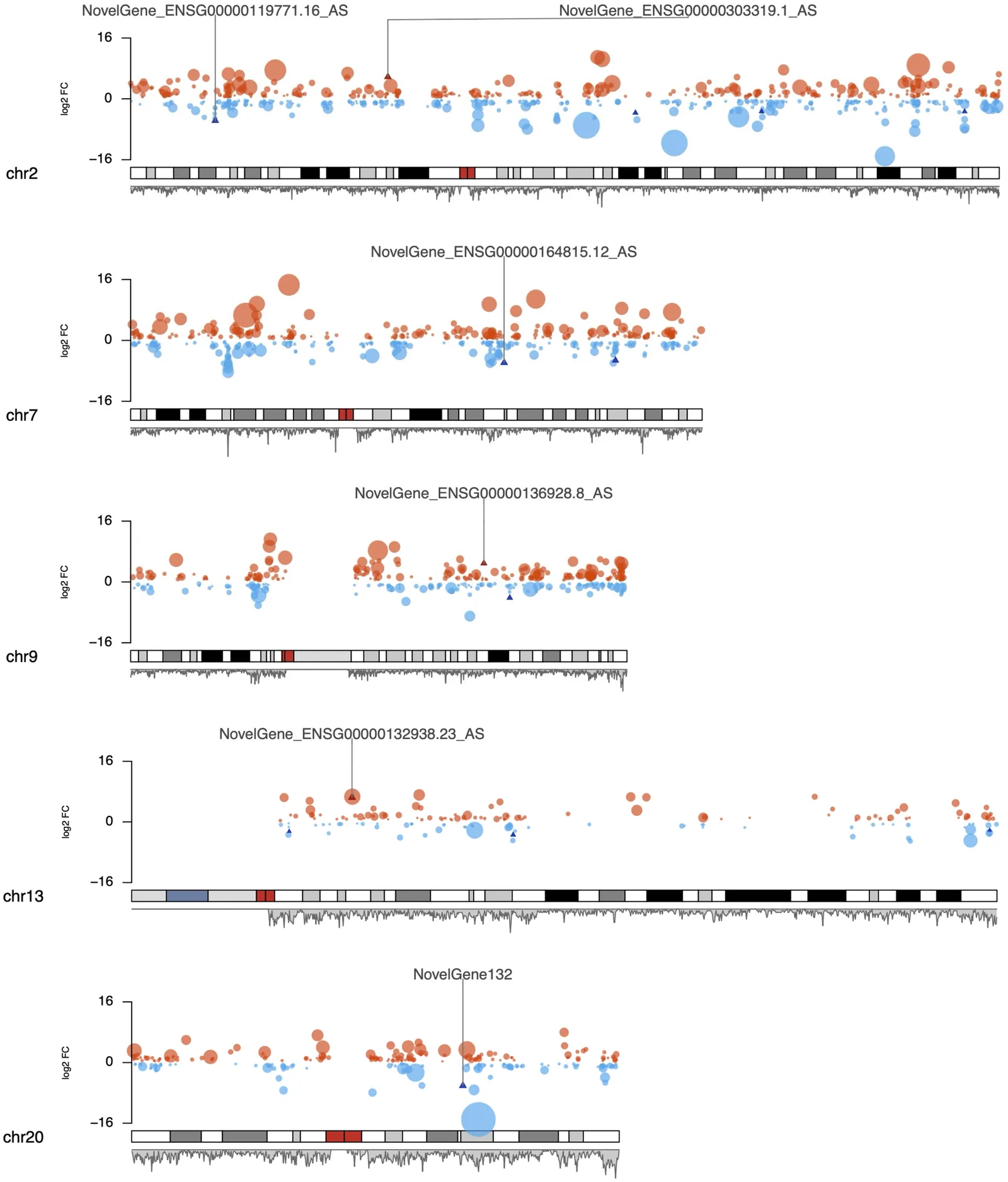
Karyotype plots representing top three upregulated and top three downregulated genes in left ventricle in comparison to skeletal muscle. For each represented human chromosome, the karyotype plot from top to bottom features the differential gene expression results, an ideogram of the chromosomes structure (bars), and a corresponding gene density plot (peaks). In the differential gene expression results panel, each gene is plotted according to its chromosomal location on the *x*-axis, and relative fold-change (log2FC) on the *y*-axis. The size of the dot corresponds to the gene's expression level. Genes with upregulated expression in the left ventricle is shown in red and those downregulated in blue. Dots represent annotated genes whilst triangles represent putative genes. The top three upregulated and top three downregulated putative genes that were significantly (*p.adj* < 0.1) differentially expressed are labelled. Chromosomes containing such genes of interest were represented in the figure.

**Figure 3:**
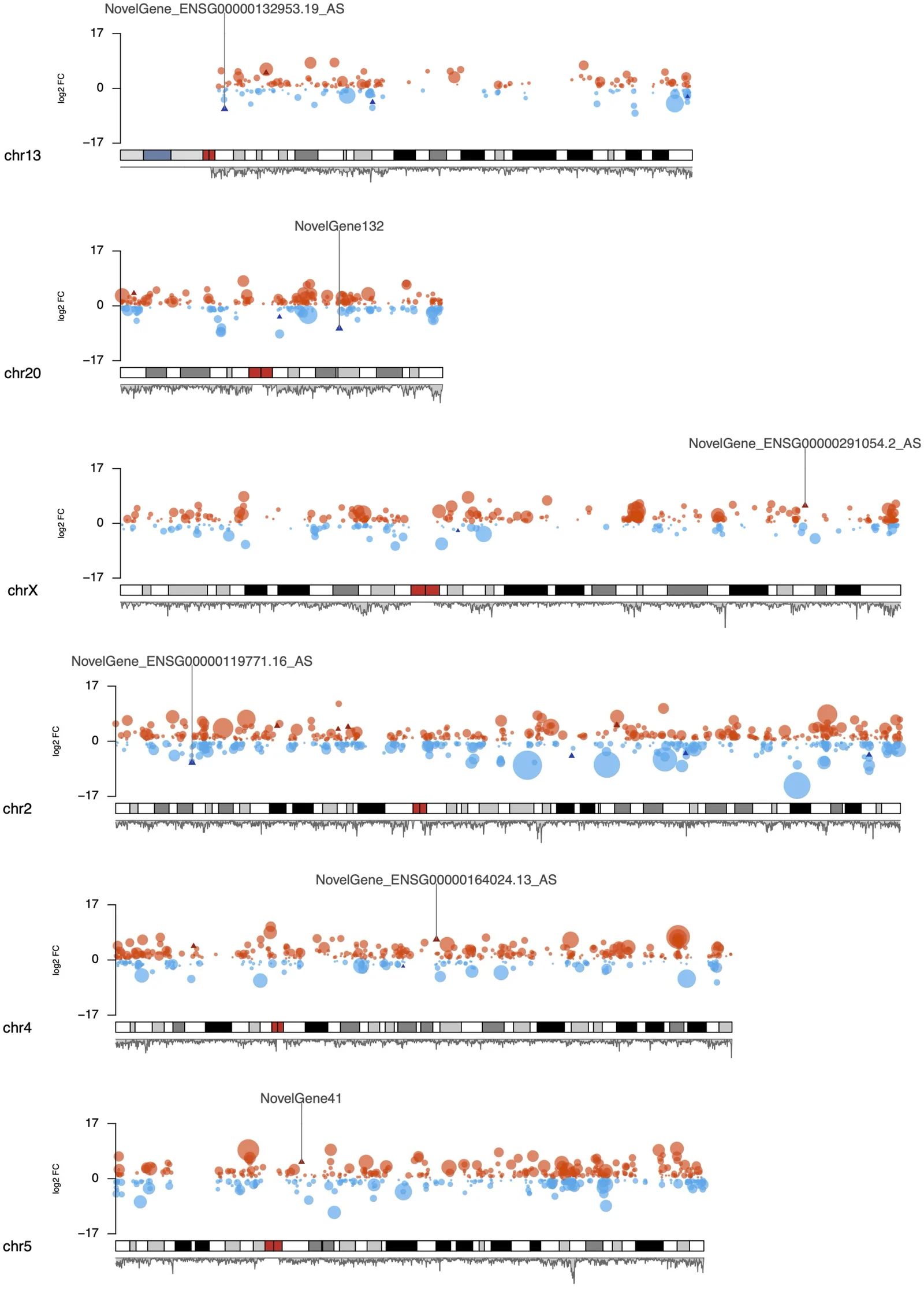
Karyotype plots representing top three upregulated and top three genes in atrial appendage in comparison to skeletal muscle. For each represented human chromosome, the karyotype plot from top to bottom features the differential gene expression results, an ideogram of the chromosomes structure (bars), and a corresponding gene density plot (peaks). In the differential gene expression results panel, each gene is plotted according to its chromosomal location on the *x*-axis, and relative fold-change (log2FC) on the *y*-axis. The size of the dot corresponds to the gene's expression level. Genes whose expression is upregulated in the left ventricle is shown in red and those downregulated in blue. Dots represent annotated genes whilst triangles represented putative genes. The top three upregulated and top three downregulated putative genes that were significantly (*p.adj* < 0.1) differentially expressed are labelled. Chromosomes containing such genes of interest were represented in the figure.

**Table 3:** Summary of the top three significantly differentially expressed putative genes.

Gene ID	Chr	Start	End	Strand	LFC	*p.adj*
Left ventricle vs. skeletal muscle						
NovelGene_ENSG00000132938.23_AS	chr13	29,105,381	29,140,343	−	6.45	1.00 × 10^−10^
NovelGene_ENSG00000303319.1_AS	chr2	71,717,260	71,743,781	−	5.78	5.58 × 10^−08^
NovelGene_ENSG00000136928.8_AS	chr9	98,488,805	98,490,275	+	4.91	6.47 × 10^−02^
NovelGene132	chr20	43,773,181	43,779,394	−	−6.09	1.25 × 10^−02^
NovelGene_ENSG00000164815.12_AS	chr7	104,133,771	104,135,836	+	−5.85	2.66 × 10^−06^
NovelGene_ENSG00000119771.16_AS	chr2	23,590,778	23,596,508	−	−5.72	3.20 × 10^−04^
Atrial appendage vs. skeletal muscle						
NovelGene_ENSG00000164024.13_AS	chr4	99,013,489	99,021,633	−	6.36	1.26 × 10^−08^
NovelGene_ENSG00000291054.2_AS	chrX	136,924,045	136,936,277	−	5.52	1.03 × 10^−04^
NovelGene41	chr5	57,403,565	57,408,051	+	5.18	1.61 × 10^−04^
NovelGene132	chr20	43,773,181	43,779,394	−	−6.99	7.68 × 10^−04^
NovelGene_ENSG00000119771.16_AS	chr2	23,590,778	23,596,508	−	−6.62	1.63 × 10^−06^
NovelGene_ENSG00000132953.19_AS	ch13	20,824,944	20,827,514	+	−6.36	9.37 × 10^−12^
Left ventricle vs. atrial appendage						
NovelGene_ENSG00000132953.19_AS	ch13	20,824,944	20,827,514	+	3.87	4.82 × 10^−03^
NovelGene_ENSG00000124523.18_AS	chr6	13,596,887	13,611,940	−	2.41	2.64 × 10^−02^
NovelGene184	KI270734.1	131,586	137,393	+	1.22	1.12 × 10^−02^
NovelGene_ENSG00000201065.1_AS	chr3	102,747,958	102,764,228	+	−4.52	2.73 × 10^−02^
NovelGene41	chr5	57,403,565	57,408,051	+	−4.19	3.83 × 10^−02^
NovelGene_ENSG00000291054.2_AS	chrX	136,924,045	136,936,277	−	−4.08	4.34 × 10^−02^

**Figure 4:**
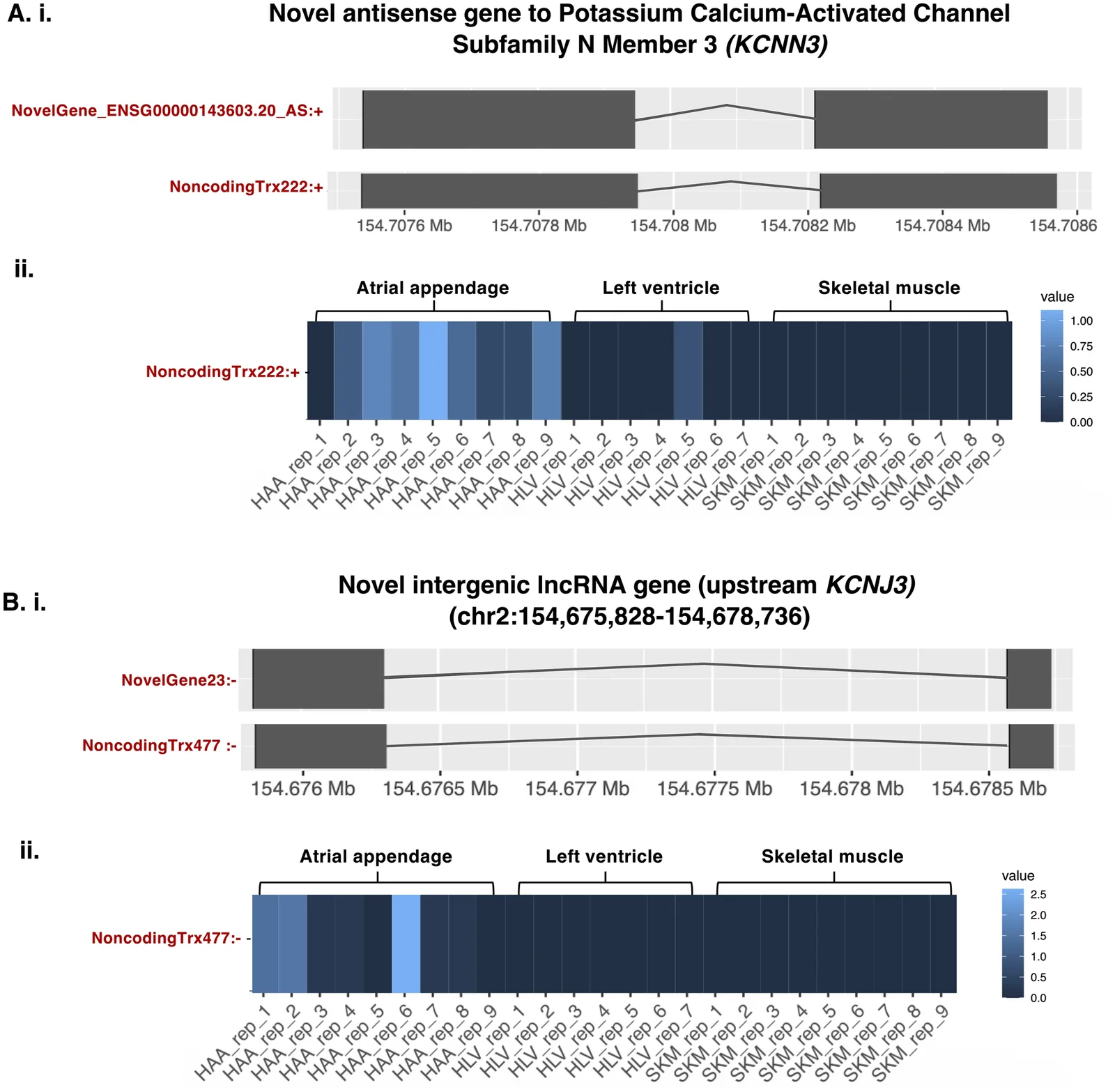
Novel antisense lncRNA gene and intergenic gene structures and expressions across heart and skeletal muscle tissues. Visualisation of the genomic structure of **(Ai)** novel antisense gene to *KCNN3* and structure of transcribed novel antisense-lncRNA, located on the sense strand. **(Bi)** The genomic structure of novel intergenic lncRNA gene NovelGene23, located on the antisense strand, and the structure of its transcribed novel lncRNA is shown. **(i)** For such structures, the genomic location (*x*-axis) is seen. Exons are indicated by grey bars with splice junction shown as the horizontal connections. A heatmap of the of the expression values, base-2 logarithmic transformations of normalised transcript expression levels [log2(CPM + 1)], is shown for all tissue replicates for **(Aii)**. NoncodingTrx222, **(Bii)**. Noncodingtrx477, and **(Cii).** NoncodingTrx285. The structure visualisation and heatmaps were generated with Bambu v.3.12.1 in an R v.4.51 environment. HAA, heart atrial appendage; HLV, heart left ventricle; SKM, skeletal muscle.

### 3.4 Cis-regulatory potential of novel lincRNA genes

To explore the potential cis-regulatory function of the 35 detected putative lincRNA genes. A Spearman's Rank Correlation test was performed on the gene expressions of the lincRNA genes in comparison to their most proximal annotated protein-coding gene. As a result, eight putative lincRNA genes were significantly correlated (FDR < 0.05) with |*rho|* values > 0.5 (Figure 5, Supplementary Table S2) and four were low to moderately correlated (FDR < 0.05) with |*rho|* values < 0.5. The expression of 14 lincRNA genes were not significantly correlated with that of their most proximal protein-coding gene, two of which were due to the lack of expression of the protein-coding gene (Supplementary Table S2). NovelGene23 is located on the antisense strand on chr chr2:154,675,828–154,678,736 bp. This intergenic gene is uniquely expressed in the atrial appendage, transcribing a lncRNA (NoncodingTrx477) that is 638 nt in length and comprised of two exons (Figure 4B). This lincRNA gene was strongly positively correlated (*rho* = 0.674; FDR = 0.00132) with the expression of KCNJ3 from which it is located 19,118 bp upstream from (Supplementary Table S2).

**Figure 5:**
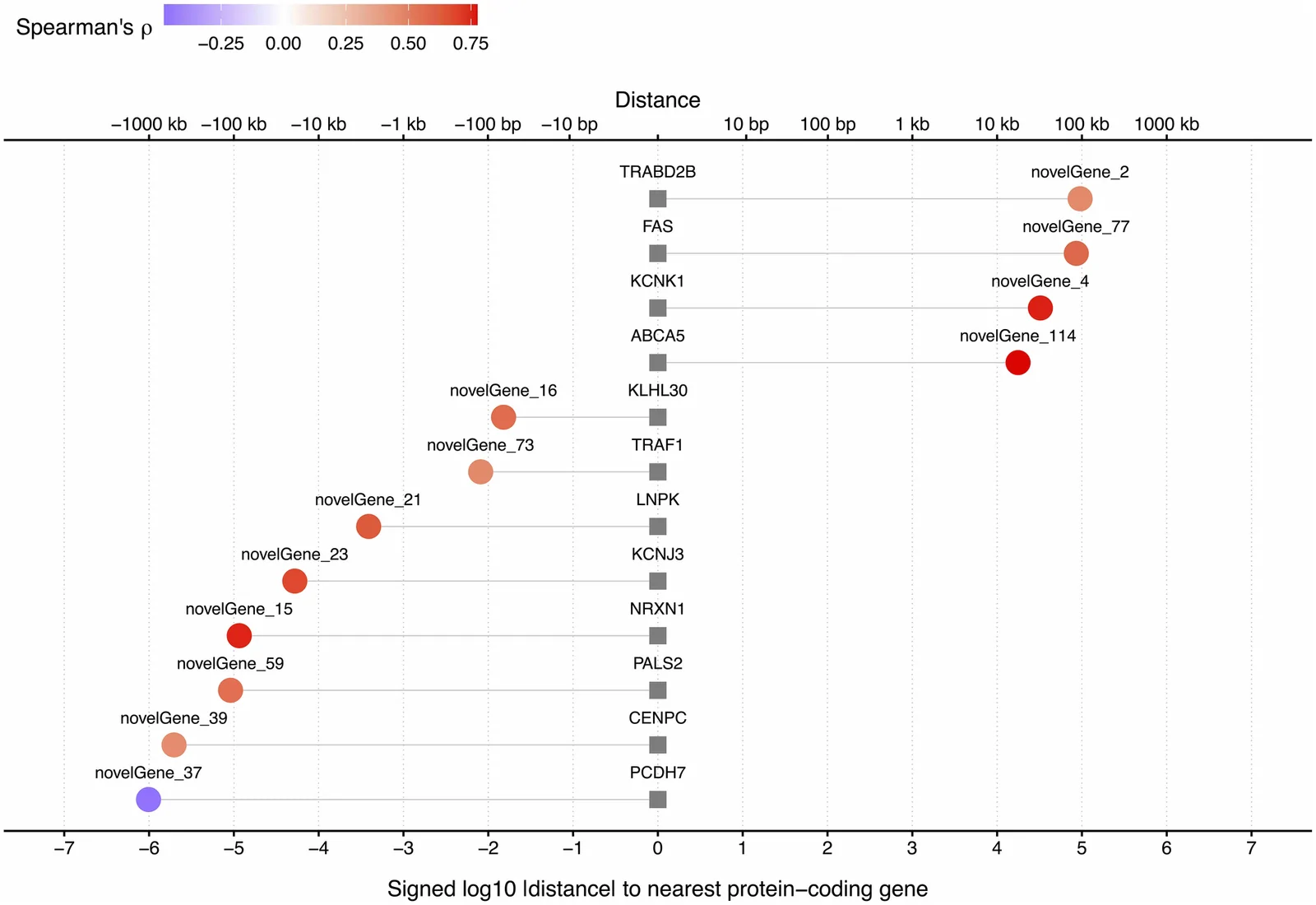
Spearman's rank correlation test of the expression patterns of putative lincRNA genes and their most proximal protein-coding gene. Putative lincRNA genes are visualised by their absolute distance and signed |log_10_| distance to the closest annotated protein-coding gene. The correlation (Spearman's *ρ*) between the putative and novel gene expression profiles across all samples are represented as indicated by the legend (*ρ* > 0, red; *ρ* < 0, blue; *ρ* = 0, white).

### 3.5 Weighted gene co-expression network analysis

Dynamic tree cutting was used to determine modules inside the gene dendrogram. As a result, 41 modules were identified. After merging modules that had a PCC greater than 75%, as indicated in Supplementary Figure S2, 29 modules remained (Supplementary Figure S3). The grey module (Module 0) contained 793 genes whose expression profiles were not correlated with any other module. Skyblue was the smallest module, containing 723 genes. The largest was the blue module (*n* = 6,376), which was significantly positively correlated with skeletal muscle (PCC = 1, *p* = 4 × 10^−27^) (Supplementary Figure S4). There was a moderate negative correlation between the blue module and both the atrial appendage (PCC = −0.57, *p* = 0.003) and the left ventricle (PCC = −0.46, *p* = 0.02). The turquoise module (nGenes = 6,625) was strongly positively correlated with the atrial appendage (PCC = 0.75, *p* = 2 × 10^−5^), not significantly correlation with the left-ventricle, and was strongly negatively correlated with skeletal muscle (PCC = −0.96, *p* = 4 × 10^−14^) (Supplementary Figure S4). The dark green module, which contained 939 genes, was significantly correlated with the left ventricle (PCC = 0.83, *p* = 3 × 10^−7^). The module was not correlated with the atrial appendage, and was moderately negatively correlated with skeletal muscle (PCC = −0.57, *p* = 0.003) (Supplementary Figure S6). The relationship between the tissues and the modules described above are exemplified in both the eigengene dendrogram and eigengene adjacency heatmap (Supplementary Figure S7).

For the set of genes belonging to the turquoise module, their turquoise module membership and significance values for the atrial appendage were moderately positively correlated (PCC = 0.49, *p* < 1 × 10^−200^) (Figure 6A). 27 putative genes were identified in this module. The putative gene with the highest atrial appendage gene significance value was novelGene_ENSG00000143603.20_AS (module membershi*p* = 0.82, gene significance = 0.78). The putative gene with the highest turquoise module membership was novelGene_163 (module membership = 0.69) gene significance = 0.83). In the dark green module, the gene dark green module membership and gene significance values of the left ventricle were also moderately positively correlated (PCC = 0.59, *p* < 3.9 × 10^−89^) (Figure 6B). This module contained 2 novel genes, with novelGene_ENSG00000124523.18_AS having the higher gene module membership (0.75) and significance value (0.73).

**Figure 6:**
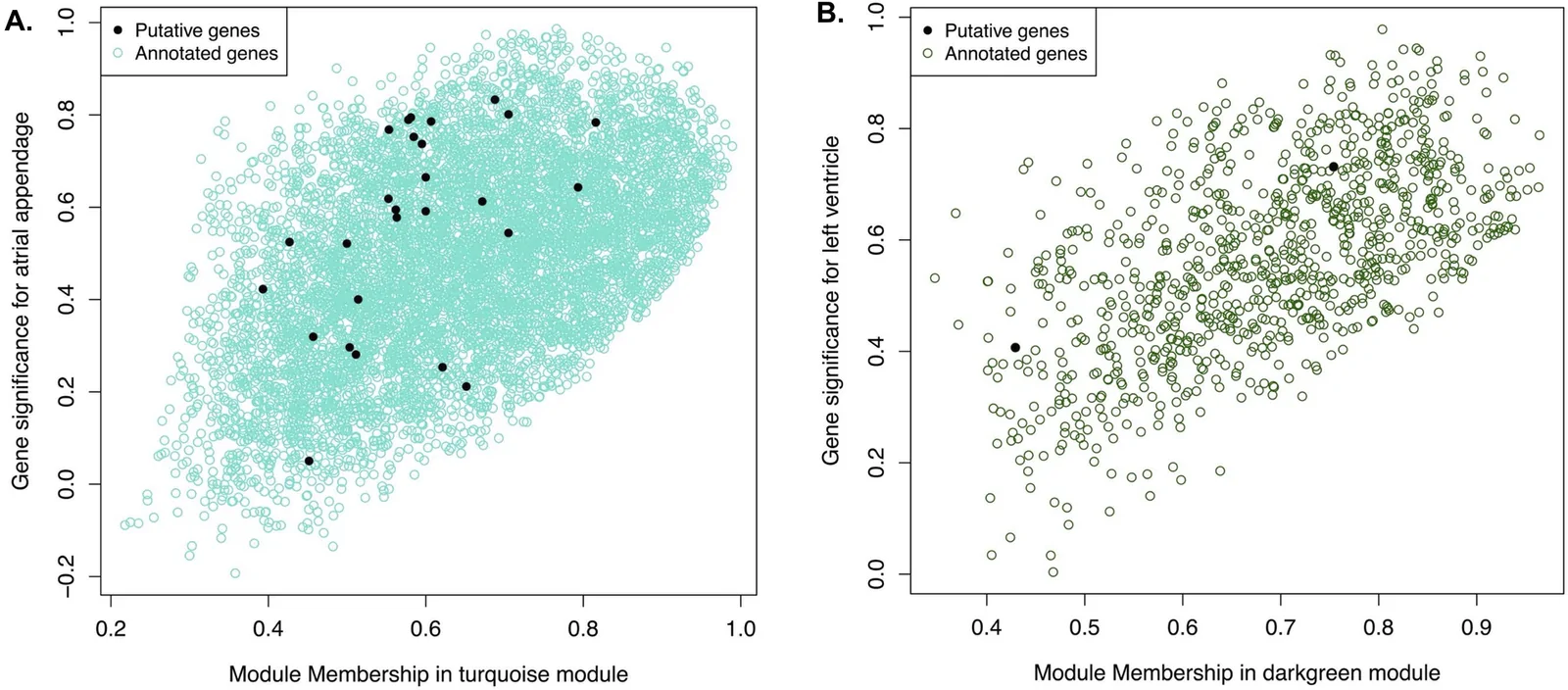
Intramodular gene module membership and significance in modules of interest. **(A)** Shown for both annotated genes (turquoise) and putative genes (black) are their module membership (*x*-axis) and gene significance (*y*-axis) in the turquoise module for the atrial appendage. **(B)** In the dark green module, shown for annotated genes (dark green) and putative genes (black) are their module membership (*x*-axis) and gene significance (*y*-axis) in the left ventricle. PCC and student asymptotic *p*-values were calculated on all genes within the modules of interest, between their association with tissue (gene significance) and their module membership.

To explore the potential functional roles of the novel genes in the turquoise module, GO and KEGG pathway enrichment analysis was performed on annotated hub genes. These hub genes are the top 10% of most connected genes (intramodular connectivity > 353, *n* = 663) in the turquoise module shown in Supplementary Figure S8. The 10 most significant biological processes (Figure 7A) and KEGG pathways (Figure 7C) enriched in the turquoise module hub genes are shown in order of their gene-ratio. The associated gene-ontology and gene relationships are visualised in concept networks for the top 10 biological processes (Figure 7B) and KEGG pathway enrichment (Figure 7D). The most significantly enriched biological process and KEGG pathway was heart contraction (GO:0060047; *p.adj* = 4.95 × 10^−19^; gene count = 46) (Figure 7A, Supplementary Table S3) and cytoskeleton in muscle cells (hsa04820; *p.adj* = 6.04 × 10^−19^; gene count = 34) (Figure 7C, Supplementary Table S4).

**Figure 7:**
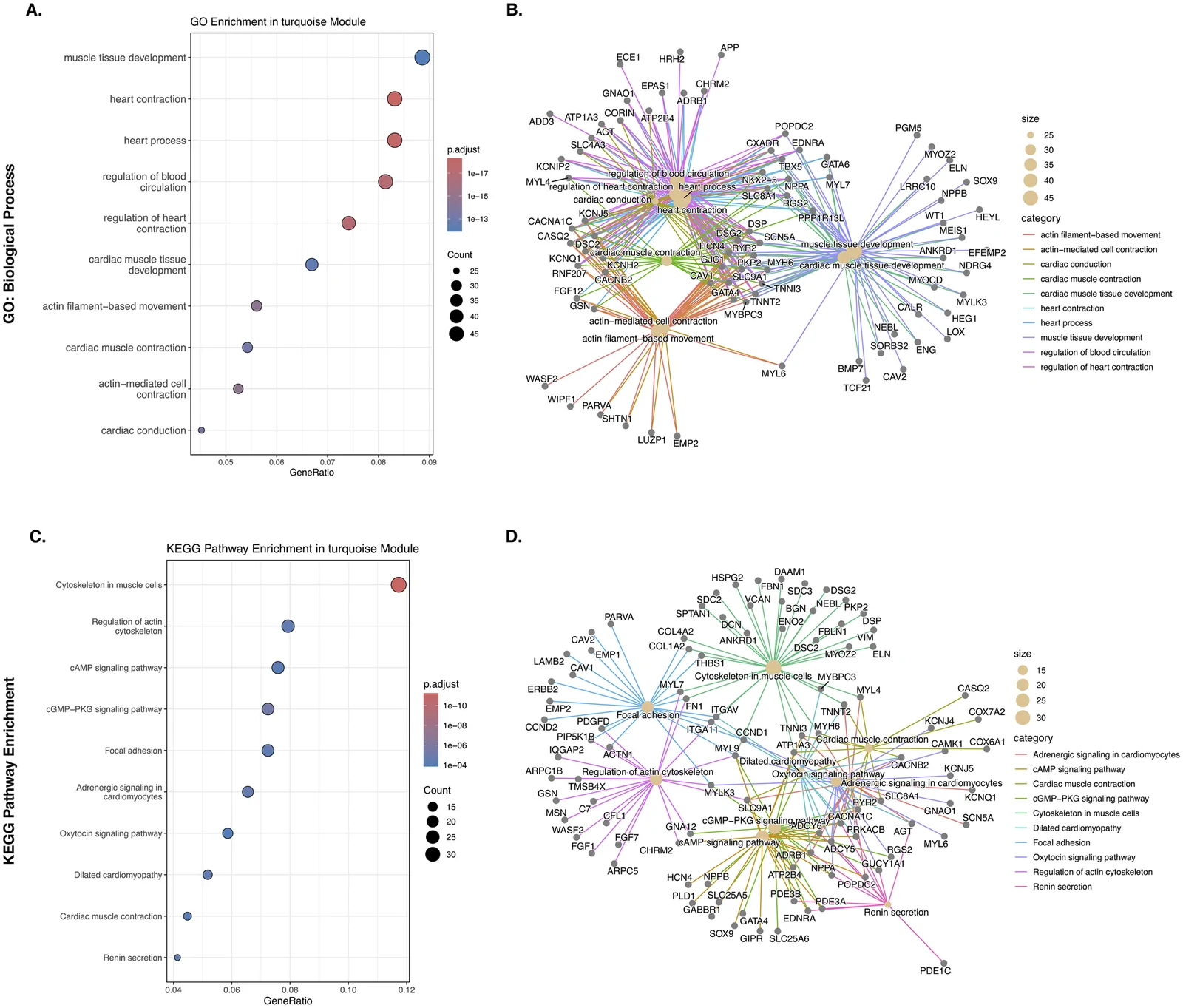
Biological process and KEGG pathway enrichment analysis of hub genes in the turquise module. The top 10 most significant (*p.adj*) **(A)** biological processes and C. KEGG pathways enriched in the turquoise module hubgenes are shown (y-axes) in terms of their gene ratio (x-axes), count, and significance (*p.adj*) as indicated in the key. The hub genes associated with the top 10 **(B)** biological processes and **(C)** KEGG pathways are visualised in concept networks.

### 3.6 Novel proteins and novel protein isoforms

Novel RNAs with protein coding potential were further characterised by the analysis of their ORFs. ORFs were first subjected to a *blastp* search against the ClusteredNR (nr_cluster_seq) database for *Homo sapiens* (taxid:9606). Out of the 142 ORFs, 62 had a significant match to a known sequence, 56 had a low match (see methods) to any known human proteins, and 24 sequences had no identified matches. The 24 ORFs with no significant match to known human protein sequences were subjected to a second *blastp* search against the same database but without the species filter. As a result, eight sequences had a low match to known protein sequences and 16 had no significant matches. ORF sequences with little to no sequence homology were utilised for downstream analyses.

### 3.7 Sequence characterisation of novel protein-coding mRNA isoforms and their ORFs

Novel mRNA isoform CodingTrx48 and lncRNA isoform NoncodingTrx273 are derived from Monoamine Oxidase B (*MAOB*) (Figure 8A). They are located on the antisense strand on chromosome X. NoncodingTrx273 features two exons and is 522 nt in length, located at chr X:43,855,661–43,882,429 bp. CodingTrx48 has four exons at chr X:43,837,512–43882429891 bp, with a length of 891 nt. Both transcripts feature one novel splice site at their second (NoncodingTrx273) and fourth (CodingTrx48) exons, seen in the extended gene annotation at the top of Figure 8A as the second and sixth exon. Both transcripts are expressed across the three tissues used in the analysis. The ORF sequence was further characterised for CodingTrx48. A prediction of the structure of the protein's peptide sequence is shown in Figure 9A, which has a predicted template modelling (pTM) score of 0.64. Scores above 0.5 are an indication of the model's similarities to the true protein structure. The predicted local distance test (plDDT) scores and expected position error (PAE) scores decrease towards the end of the sequence, where the model would vary the most from the true structure. The results of further sequence analysis of the ORF are summarised in Supplementary Figure S9A. No signal peptides or transmembrane regions were predicted based on the consensus. The peptide/protein encoded by the ORF is homologous to the human Monoamine Oxidase-B (MAO-B) protein [also known as amine oxidase (flavin-containing) B] [Protein Data Bank (PDB) ID: 4a79A] at regions corresponding to annotated exons and bears no homology to transmembrane proteins at the region corresponding to the unannotated 4th exon. Similarly, protein domains, conserved sequence domains, and *blastp* matches are all identified for the MAO-B protein at the regions corresponding to the first three annotated exons (Supplementary Figure S9A).

**Figure 8:**
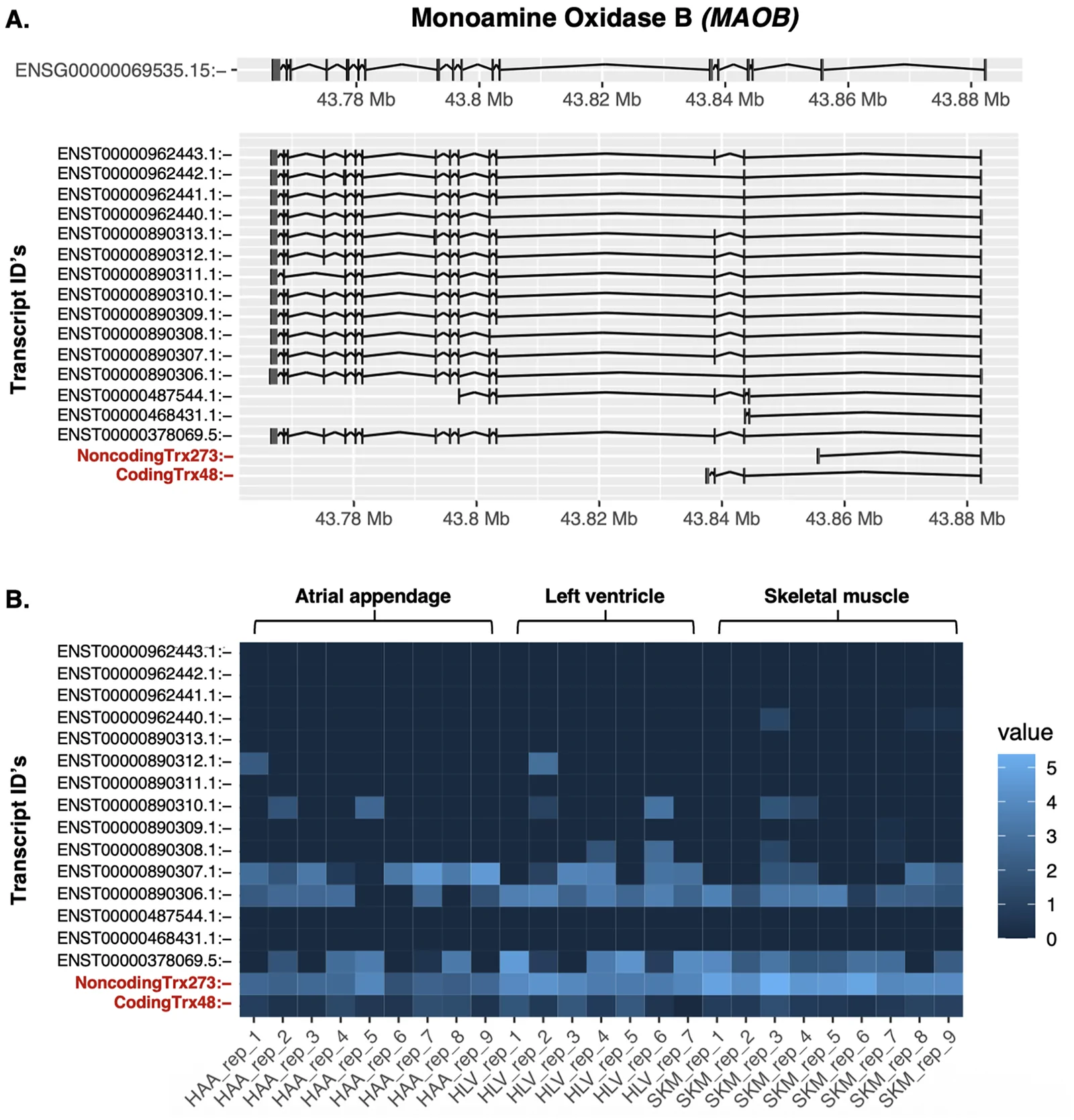
Novel RNA isoforms CodingTrx48 and NoncodingTrx273 structure and expression across heart and skeletal muscle tissues. **(A)** Visualisation of the structures of annotated transcriptional isoforms alongside novel mRNA isoform CodingTrx48 and novel lncRNA NoncodingTrx273 encoded by monoamine oxidase B (*MAOB*), located on the antisense strand. For such structures, the genomic location (*x*-axis) is shown. Exons are indicated by grey bars with splice junction shown as the horizontal connections. **(B)** A heatmap of the of the expression values, base-2 logarithmic transformations of normalised transcript expression levels [log2(CPM + 1)], The structure visualisation and heatmaps were generated with Bambu v.3.12.1 in an R v.4.51 environment. HAA, heart atrial appendage; HLV, heart left ventricle; SKM, skeletal muscle.

**Figure 9:**
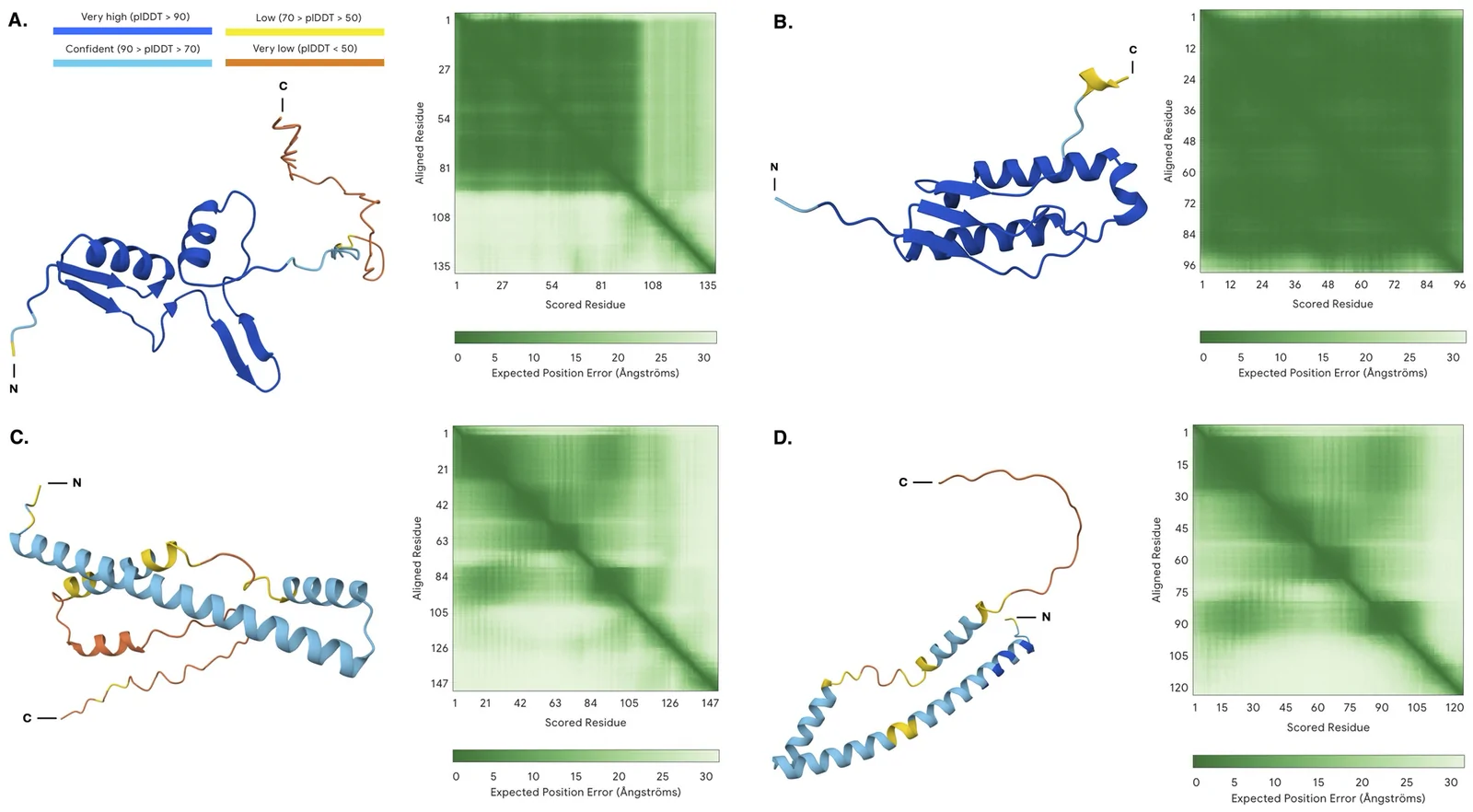
Alphafold3 structure prediction of proteins encoded by novel mRNAs. Alphafold3 protein structural prediction of the ORFs of **(A)** CodingTrx48 (pTM = 0.64), **(B)** CodingTrx63 (pTM = 0.89), **(C)** CodingTrx87 (pTM = 0.36) and **(D)** CodingTrx79 (pTM = 0.35). The plDDT score ranges for individual amino acid residues are on the protein structure. **(A–D)** The pTM score produced for the AlphaFold structures is an overall score of model accuracy, where values over 0.5 suggest the prediction is similar to the true structure of the protein. The expected position error (PAE) scores are shown for residue pairs from the protein model, where values close to zero (dark green) indicate a lower chance of error. pTM; predicted template modelling, plDDT; predicted local distance difference test.

Novel mRNA isoform CodingTrx63 of NAD Synthetase 1 (*NADSYN1*) is visualised alongside annotated transcripts of the same gene (Figure 10A). CodingTrx63, located on the sense strand of chr 11:71,453,199–71,460,947. It has four exons with an overall length of 587 nt, and is a new combination of known splice sites. Specifically, the mRNA bears similarity to the genomic structure of ENST00000525245.1 with an exon-skipping event on the fourth exon (Figure 10A). The mRNA is expressed across all tissues, with an elevated expression in the atrial appendage and skeletal muscle (Figure 10B). The Alphafold3 protein structural prediction of the ORF of CodingTrx63 is shown in Figure 9B. The model has a pTM score of 0.89. This score suggests the predicted structure would be similar to the true protein structure (Figure 9B). The plDDT score ranges for individual AA residues are on the protein structure. Additionally, across almost all AA residue pairs, the PAE scores are close to zero, indicating a very low chance of error in the spatial arrangement of the structure. The high plDDT scores also support the strength of this prediction, only slightly falling towards the C-terminus where the exon-skipping event takes place. Summarised in Supplementary Figure S9B are the results of TOPCONS topology prediction, InterProScan protein domain prediction, associated PANTHER gene ontology terms, *blastp* results, and NCBI's conserved domain search results for CodingTrx63 shows that the ORF sequence bears significantly homology to the Glutamine-dependent NAD(+) synthetase protein encoded by *NADSYN1*.

**Figure 10:**
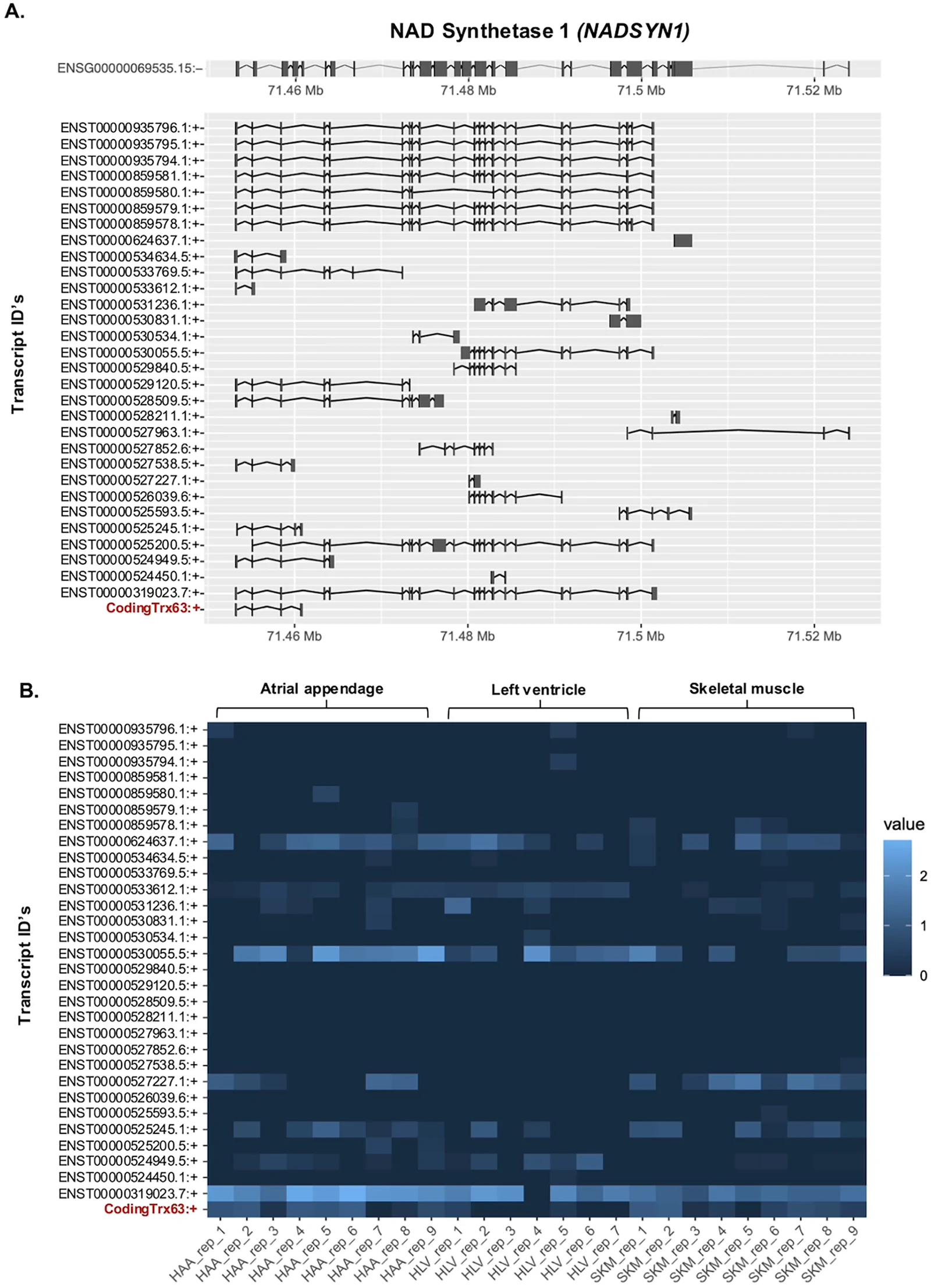
Novel mRNA isoform CodingTrx63 structure and expression across heart and skeletal muscle tissues. **(A)** Visualisation of the structures of annotated transcriptional isoforms of NAD synthetase 1 (*NADSYN1*) alongside novel mRNA isoform CodingTrx63, located on the sense strand. For such structures, the genomic location (*x*-axis) is shown. Exons are indicated by grey bars with splice junction shown as the horizontal connections. **(B)** A heatmap of the of the expression values, base-2 logarithmic transformations of normalised transcript expression levels [log2(CPM + 1)], is shown for all tissue replicates. The structure visualisation and heatmaps were generated with Bambu v.3.12.1 in an R v.4.51 environment. HAA, heart atrial appendage; HLV, heart left ventricle; SKM, skeletal muscle.

Novel mRNA isoforms CodingTrx79 and CodingTrx87 of *KCNK15* are located on the sense strand of chr 20:44,745,847–44,747,201 and chr 20:44,745,838–44,749,759 respectively (Figure 11A). Both CodingTrx79 and CodingTrx87 are comprised of two exons, where the second exon of both structures are not present in the only known transcript of KCNK15. CodingTrx79 is 652 nt in length and CodingTrx87 is 501 nt. The three transcripts have variable expressions across the atrial appendage, left ventricle, and skeletal muscle tissues as shown in Figure 11B. The predicted protein structures of the ORFs are shown for CodingTrx87 (Figure 9C) and CodingTrx79 (Figure 9D) where the pTM scores are notably poor (CodingTrx87, pTM = 0.36; CodingTrx79, pTM = 0.35). Despite the poor confidence of the model structures, the prediction of alpha-helices is well supported by both PAE and plDDT scores. Toplogy prediction by ORF sequence analysis with TOPCONS of both novel mRNAs revealed signal peptides at the N-terminals (Supplementary Figure S10). Additionally, both ORFs displayed homology to transmembrane protein human two pore domain potassium ion channel TREK2 [Protein Data Bank (PDB) ID: 4bw5A], which is derived from the potassium two pore domain channel subfamily K member 10 (*KCNK10*) gene. Although the TOPCONS consensus predictions do not show any transmembrane regions (Supplementary Figure S10), the ΔG values decrease at regions of the ORF sequence with homology to transmembrane regions of TREK2, suggesting there may be an energetically favoured membrane insertion at such locations for both CodingTrx79 and CodingTrx87 (Supplementary Figure S10). To further evaluate this, the membrane insertion energies of CodingTrx87 and CodingTrx79 was compared to those of known soluble and transmembrane proteins (Supplementary Figure S1^1^) As shown in Supplementary Figure S1^1^, both ORFs have membrane insertion energies within the range of known transmembrane proteins. The prediction of the proteins' location within the plasma membrane by QMEANBrane is also shown (Figure 12). To summarise, the results of all sequence analyses of the ORFs of CodingTrx48 and CodingTrx79 are shown in Supplementary Figure S9C,S9D respectively. The results of TOPCONS topology prediction, InterProScan protein domain prediction, associated PANTHER gene ontology terms, *blastp* results, and NCBI's conserved domain search are displayed. Notably, both structures have two voltage-gated potassium channel domains as part of the two-pore domain potassium channel. Additionally, CodingTrx79 has a conserved ion transport channel located at the second voltage-gated potassium channel domain.

**Figure 11:**
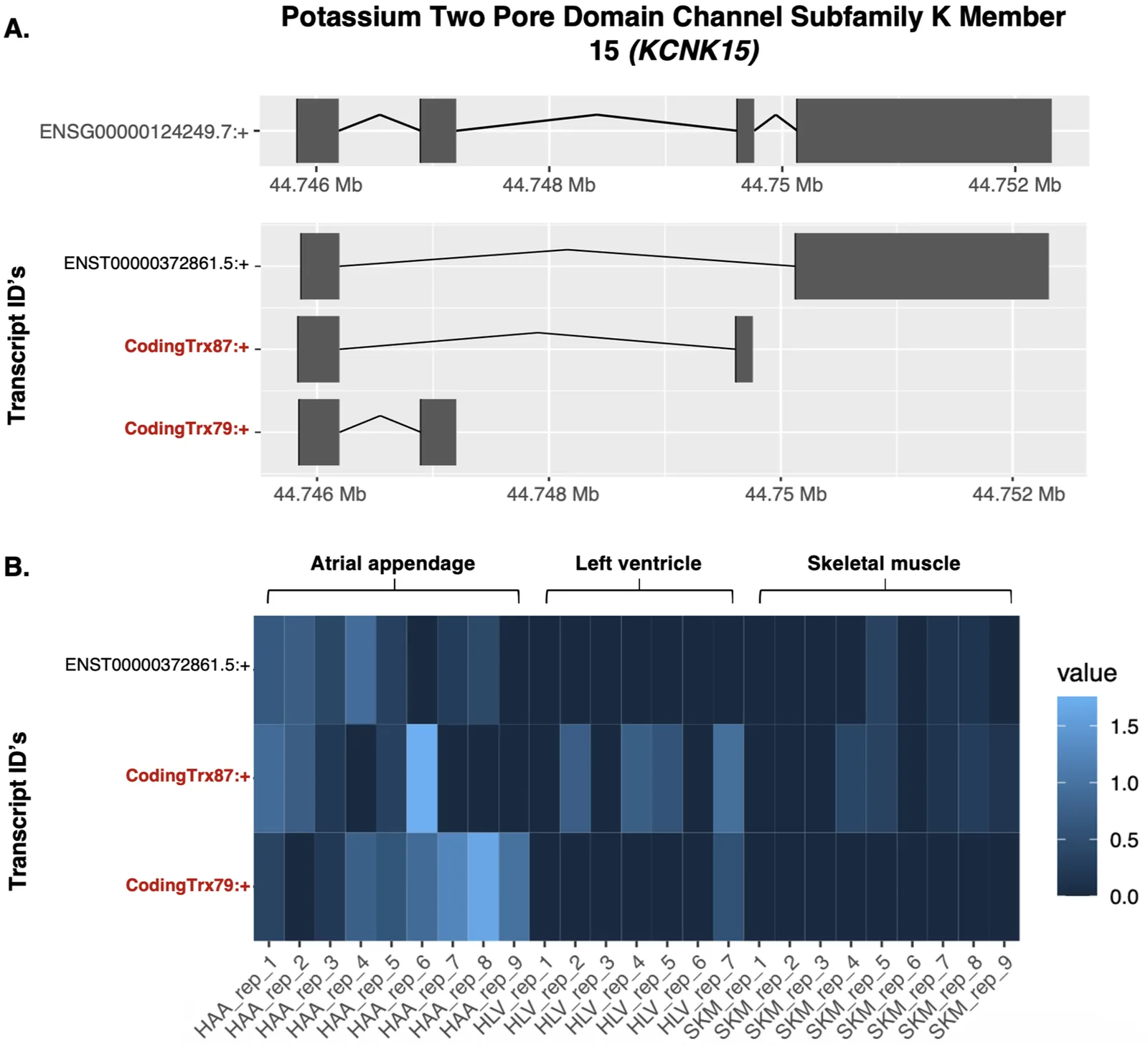
Novel mRNA isoforms CodingTrx87 and CodingTrx79 structure and expression across heart and skeletal muscle tissues. **(A)** Visualisation of the structures of annotated transcriptional isoforms of potassium two pore domain channel subfamily K member 15 (*KCNK15*) alongside novel mRNA isoforms CodingTrx87 and CodingTrx79, located on the sense strand. For such structures, the genomic location (*x*-axis) is shown. Exons are indicated by grey bars with splice junction shown as the horizontal connections. **(B)** A heatmap of the of the expression values, base-2 logarithmic transformations of normalised transcript expression levels [log2(CPM + 1)], is shown for all tissue replicates. The structure visualisation and heatmaps were generated with Bambu v.3.12.1 in an R v.4.51 environment. HAA, heart atrial appendage; HLV, heart left ventricle; SKM, skeletal muscle.

**Figure 12:**
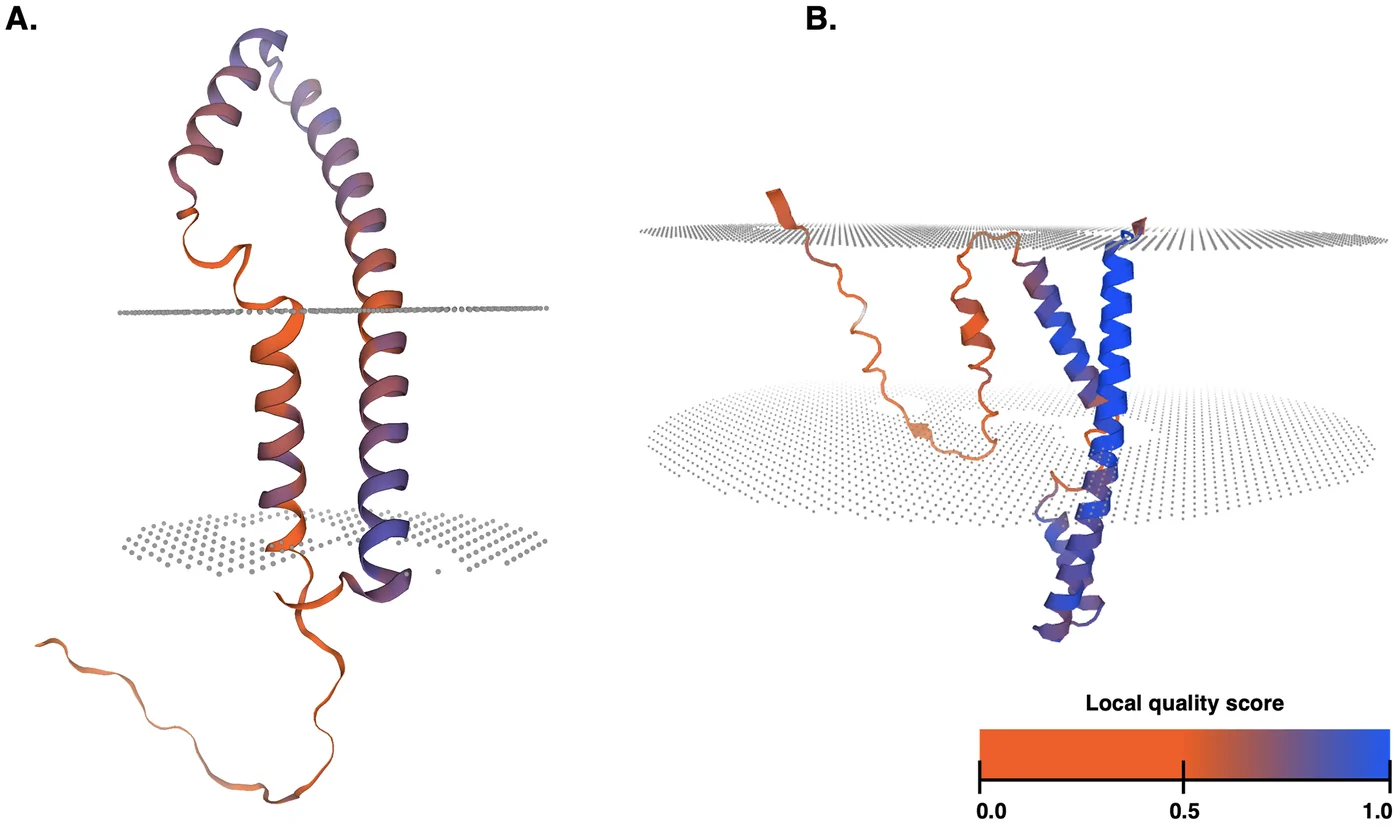
Membrane location of proteins encoded by CodingTrx87 and CodingTrx79. Prediction of membrane location based on structural information using the solvation model by QMEANBrane v4.5.0. Membranes are shown for the **(A)** CodingTrx87 and **(B)** CodingTrx79 Alphafold3 predicted protein structures. The protein structure is coloured according to the local quality score of amino acids calculated by the QMEANBrane scoring function, where values closer to one indicate a higher local quality.

## 4 Discussion

Alternative splicing is a heavily regulated cellular mechanism and is one of the key transcriptomic factors in healthy cardiac development and function. Changes in the relative abundance of individual RNA isoforms can define the differences between cardiomyocytes of different tissue origin. This is also observed at various stages of development and in numerous cardiovascular diseases and pathologies (15). The analysis of SRS transcriptomic data to detect, quantify, and characterise such RNA isoforms can be challenging, especially at complex loci, large genes, and short tandem repeats. The computational task of not only correctly determining the structure of these RNA isoforms, which can be similar in composition, but also accurately measuring their relative abundance contributing to the measure of gene expression is erroneous due to the short read lengths of the fragmented RNA. LRS proposes a solution to this task, as RNA fragmentation is not required prior to sequencing, resulting in read lengths up to the entire length of native transcripts. Such reads can then be better assigned to the various transcripts, significantly improving isoform expression measurements. The primary aim of this study was to apply this principal to a selection of prized GTEx samples that were recently re-sequenced with ONT's nanopore sequencing (36). In the study by Glinos et al. (36) FLAIR was employed for transcriptome assembly (68). The biases raised from the selection of a long-read transcriptome assembler were recently detailed by the LRGASP study (28). In our previous work, it was also demonstrated that assembly using FLAIR in comparison to Bambu and IsoQuant yielded a lower proportion of isoforms across the NNC, genic-genomic, antisense, and fusion structural categories after the removal of artifactual transcripts (14). Taking this into consideration during the analytical pipeline construction, as well as the final recommendations proposed by the LRGASP study for accurate identification of novel splice isoforms, we revisited this dataset to report novel features not previously described by the original study (36). The described computational pipeline also incorporates several measures to ensure the inclusion of artefactual novel isoforms is minimised, as detailed in the methodology (Section 2.3, 2.6–2.7).

As a result, 491 novel transcriptional isoforms derived from cardiac and skeletal muscle tissues were robustly detected, quantified, and computationally characterised, contributing to the overall annotations of human tissues. In addition to isoforms derived from annotated genes, 173 novel isoforms derived from putative genes have been detected. This is comprised of 54 intergenic and 112 antisense putative genes. The putative genes and RNA isoforms were further examined to determine potential functional relevance in the cardiac transcriptome. This was conducted with a specific focus on putative genes uniquely expressed in cardiac tissues, and those detected in gene co-expression networks significantly correlated with either the atrial appendage or left ventricle. 44 putative genes were significantly upregulated in cardiac tissues in comparison to skeletal muscle, and 42 were uniquely expressed in one (*n* = 14) or two (*n* = 28) of the analysed heart tissues. Furthermore, putative genes were identified in modules significantly positively correlated with the atrial appendage (*n* = 27) and the left ventricle (*n* = 2) by WGCNA. Hub genes within the module correlated with the atrial appendage demonstrated cardiac-specific biological processes and KEGG pathways, supporting the notion that the 27 putative genes within this module may bear similar functionalities (Figure 7). At the transcriptome level, new RNA isoforms derived from annotated genes with known roles in cardiac physiology and/or pathophysiology were detected. Additionally, there were several novel mRNA isoforms (not suspected to be sensitive to nonsense mediated mRNA decay) that were determined to encode new protein isoforms which retain annotated protein domains, conserved domains, binding sites, signal peptides, and transmembrane domains associated with functional aspects of the cardiac transcriptome. Such isoforms were presented in more detail as examples from the total set curated by this study, which are described below.

The novel antisense gene NovelGene_ENSG00000143603.20_AS and lncRNA isoform NoncodingTrx222 are antisense to the protein-coding gene potassium calcium-activated channel subfamily N member 3 (*KCNN3*) (69). (Figures 4Ai). The gene is significantly upregulated in the atrial appendage in comparison to skeletal muscle (Figures 4Aii). *KCNN3* encodes small conductance calcium-activated potassium channel protein 3 (SKCa3), which is part of a family of SK channels. Such channels are expressed in excitable tissues, namely neurons and cardiomyocytes, with a known increased expression in the atria in comparison to the ventricles (70, 71). Genome-wide association studies have determined that KCNN3 is a susceptibility locus for atrial fibrillation (72), a cardiac disorder of supraventricular arrhythmia. Overexpression of SK3 has demonstrated correlation with shorter action potential duration (73). Antisense lncRNAs are non-coding RNAs that are both greater than 200 nt and transcribed from the opposing strand of a protein-coding gene. They can modulate the expression of their protein-coding counterparts by forming RNA-DNA complexes or double-stranded RNA structures. As NoncodingTrx222 also demonstrates an increased expression in atrial tissue (Figures 4Aii), the function of this molecule could be to modulate the expression of KCNN3. However, this potential function should be further examined to determine whether the lncRNA influences the expression or alternative splicing of its protein-coding counterpart. As shown in Supplementary Figure S1^2^, the splice junctions of this putative gene can be seen in the corresponding SRS dataset, but the short reads surrounding this genomic location produced from KCNN3 generates sequence noise that makes it hard to detect with short-read based computational methods. Seen in the raw reads of the LRS dataset, the splice junction and exons of this locus are far clearer and more consistent, which enables its detection and abundance quantification in this study.

LincRNA genes demonstrate potential for cis-regulation of nearby protein-coding genes. We assessed this regulatory potential for the 35 detected putative lincRNA genes by performing a Spearman's Rank Correlation between their gene expression profiles across the 25 samples in comparison to that of the most proximal annotated protein-coding gene. Eight novel lincRNA genes were significantly moderately correlated (FDR < 0.05; |*rho|* values > 0.5) (Figure 5, Supplementary Table S2). Notably, NovelGene23 which was uniquely expressed in the atrial appendage, was strongly positively correlated with the expression of Potassium Inwardly Rectifying Channel Subfamily J Member 3 (*KCNJ3*) (*rho* = 0.67, *p.adj* = 0.0013). The novel intergenic gene NovelGene23 is uniquely expressed in the atrial appendage, transcribing lncRNA NoncodingTrx477 (Figures 4Bii). The same observations of the SRS and LRS generated reads from NovelGene_ENSG00000143603.20_AS can be made for NovelGene23 as well, as shown in Supplementary Figure S1^3^. *KCNJ3* encodes the G protein-gated inwardly rectifying potassium channel (Kir3.1) (74). This protein forms an acetylcholine-activated potassium channel with the Kir3.4 protein encoded by *KCNJ5* (75). *KCNJ3* is primarily expressed in the heart, with its protein complex functioning in cardiac repolarisation and heartbeat regulation. Nonsynonymous mutations in the *KCNJ3* gene have been associated with hereditary bradyarrhythmia's (75). As NovelGene23 is located upstream of *KCNJ3* encoding a lincRNA molecule with it's expression strongly positively correlated to that of *KCNJ3*, there is a potential for cis-regulation. However, only a small number of tissue types were analysed in this study so the expression of this putative gene across other tissue/cell types should be determined. Additionally, this cis-regulatory potential would have to be confirmed experimentally as this is just a prediction based on co-expression.

Novel mRNA isoform CodingTrx48 and lncRNA isoform NoncodingTrx273 are derived from *MAOB* (Figure 8A). CodingTrx48 is structurally classified as NNC, as it features one novel splice site at the fourth exon. Similarly, NoncodingTrx273 is also NNC, as it contains a novel splice site at the second exon (Figure 8A). Both transcripts are expressed across the atrial appendage, left ventricle, and skeletal muscle (Figure 8B). The translated amino acid sequence of CodingTrx48's ORF is also homologous to the human MAO-B (Supplementary Figure S9A). MAO-B is localised to various regions of the mitochondria, such as in the intracellular region, or in its membrane-bound form, to the outer mitochondria membrane. The primary role of the flavin monoamine oxidase family is catalysing oxidation of various amine substrates such as polyamines and modified AAs (76). They contain conserved flavin adenine dinucleotide (FAD) binding domains (76, 77), which were conserved in CodingTrx48 alongside the conserved nicotinamide adenine dinucleotide phosphate (NADP+) binding domain (Supplementary Figure S9A). MAO-B has been studied extensively for its major role in contributing to the pathogenesis of various neurodegenerative disease such as Parkinson's and Alzheimer's disease, as it metabolises the tertiary amine neurotoxin 1-methyl-4-phenyl-1,2,4,5-tetrahydropyridine (MPTP) which causes neuronal loss (78). MAO-B not only assists in the metabolism of neuroactive amines in the central nervous system, but also vasoactive amines in various peripheral tissues. As a result of this, monoamine oxidases have been examined for their contribution in cardiovascular pathophysiology (79, 80). Both major protein isoforms, MAO-A and MAO-B, are expressed in cardiomyocytes and heart tissues, with MAO-A displaying a moderately higher expression (81). In the heart, oxidative stress by monoamine oxidases is a known contributor of cell death, mitochondrial dysfunction, and maladaptive cardiac hypertrophy via generation of hydrogen peroxide, which can lead to heart failure (80). It has been demonstrated that monoamine oxidase inhibition can prevent oxidative stress and thus reduce cardiac dysfunction, which may be beneficial for the treatment of cardiac pathologies (80). By stringently analysing this LRS dataset, two new exons of the well characterised *MAOB* gene have been determined, expanding the current annotations. This is elucidated in the raw reads of the LRS sample, which demonstrates the notion that longer reads allow for more accurate transcriptome assembly and quantification in comparison to SRS methods (Supplementary Figure S1^4^). Sequence analysis with TransDecoder2 (via SQANTI-qc) detected an intact CDS for the longer novel transcript of *MAO-B* (CodingTrx48). By bioinformatically characterising the predicted ORF of this transcript, several features of the MOA-B protein were retained for almost the entire ORF sequence, excluding the part of the sequence pertaining to the last (new) exon. This included protein domains, conserved domains, and binding sites for NADP and FAD (Supplementary Figure S9A). It cannot be determined using sequence inspection alone whether this protein isoform may be a fraction of a larger protein isoform, so it is recommended that this be evaluated using techniques such as RT-qPCR and mass spectrometry for both the evidence of translation and realisation of the full protein isoform.

Novel mRNA isoforms CodingTrx79 and CodingTrx87 are derived from potassium two pore domain channel subfamily K member 15 (*KCNK15*) (Figure 11). They are both NNC transcripts, each containing an unannotated second exon. *KCNK15* has one known mRNA isoform, ENST00000372861.5. Additionally, the SRS sample supports the splice junctions present in the novel RNA isoforms of the LRS samples (Supplementary Figure S1^5^). All three mRNA isoforms are expressed across the atrial appendage, left ventricle, and skeletal muscle tissues (Figure 11B). *KCNK15* encodes a potassium channel protein that is currently known to not form a functional plasma membrane K+ current, at least not on its own (82). During myocardial infarction, the myocardial tissues suffer from hypoxia and ischemia (83). Once blood flow is restored, the cardiomyocytes can suffer from myocardial ischemia-reperfusion injury (MIRI), which causes a cascade of pathophysiological reactions that may result in arrythmia or heart injury (83, 84). Potassium ion channels have a significant role in MIRI, including two-pore domain potassium channels like *KCNK15,* which has been extensively reviewed by Zhang et al. (83). The role of *KCNK15* and its encoded protein TASK-5 has not been clearly demonstrated in the literature. Topology prediction of the ORF sequence of CodingTrx79 and CodingTrx87 with TOPCONS revealed signal peptides at the N-terminus, which are known to have important roles in transmembrane transport, protein synthesis, and intracellular signal transduction (85) (Supplementary Figure S10). Both ORFs also had homology to transmembrane protein TREK2 encoded by *KCNK10* (Supplementary Figure S10). The results of further sequence analysis support the notion that the TASK-5 protein encoded by the current incomplete annotation of *KCNK15* may just be a fragment or subunit of a larger protein complex, and that a larger protein isoform encoded by *KCNK15* may form a function potassium channel. This is supported by the detection of a conserved ion transport protein domain in CodingTrx79 (Supplementary Figure S9D). This is further elucidated by the topological prediction of CodingTrx79 and CodingTrx87 by QMEANBrane, which shows both predicted protein structure to be imbedded in the cell membrane (Figure 12). It is recommended that PCR amplification and Sanger sequencing of this region should be performed to confirm such predictions, and techniques such as mass spectrometry and x-Ray crystallography be utilised to realise the full protein structure.

The novel mRNA isoform CodingTrx63 of *NADSYN1* is a new combination of known splice sites, seen as an exon-skipping event at the fourth exon of ENST00000525245 (Figure 10A). The mRNA is expressed across all tissues, with an increased expression in the atrial appendage and skeletal muscle (Figure 10B). Additionally, the predicted ORF shows significant homology to the Glutamine-dependent NAD(+) synthetase protein encoded by *NADSYN1*. This protein catalyses the last reaction of *de novo* NAD synthesis. Mutations in this gene have been implicated in cardiac pathologies including Vertebral, Cardiac, Renal, And Limb Defects Syndrome 3 (VCRL3) (86–88) and Congenital Vertebral-Cardiac-Renal Anomalies Syndrome, also known as Congenital Nad Deficiency Disorder (89). As seen in Supplementary Figure S9B, protein domains and conserved domains of the annotated protein were retained in this isoform. This demonstrates the effectiveness of LRS coupled with the utilised bioinformatics pipeline at discovering alternatively spliced mRNA isoforms in comparison to traditional SRS-based methods. This can be seen in the raw reads of the SRS sample and LRS sample in Supplementary Figure S1^6^, where although the exon skipping event can be seen in one read of the raw SRS dataset, its structure and abundance in comparison to other RNA isoforms of the same gene in the LRS sample is explicit, allowing for its detection in computational methods.

### 4.1 Limitations and future directions

The analysed long-read RNA-seq dataset was derived from nanopore sequencing of total RNA isolated from bulk tissue extractions from the atrial appendage, left ventricle, and skeletal. Thus, it cannot be determined if the novel features are specific to the tissue or to a particular cell type. Further experiments utilising single-cell sequencing technologies or fluorescence *in situ* hybridisation could be conducted to determine the cell specificity and localisation of these novel isoforms, which could also aid in elucidating their functions (90). As the analysis was performed on just three tissues, the expression of these isoforms in tissues of other systems is yet to be determined, which could be achieved by repeating the methodology on a larger dataset. Larger sample sizes for individual tissues and incorporation of samples from a wider population cohort would also improve the reliability of results. It should also be noted that despite the inclusion of multiple tools and filters to improve the reliability of putative gene and novel RNA discovery, these are bioinformatic predictions are are yet to be experimentally validated. Therefore, it is imperative that further analyses are undertaken to confirm such features through targeted RT-qPCR amplification and Sanger sequencing. For protein-coding mRNAs, evidence for their translation should be confirmed. The reliability of novel proteins and protein isoforms could be supported by the analysis of mass spectrometry data for peptide hits, however the evidence for this at the isoform level is typically very low as the abundance of peptides that can be assigned to individual isoforms is generally low (91). Future studies could employ techniques such as x-ray crystallography to validate the protein structures at atomic resolution as the accuracy of computational *ab initio* structural predictions can be low.

### 4.2 Concluding remarks

Overall, this study has employed a stringent analytical pipeline for the analysis of nanopore cDNA sequencing data to identify putative genes, RNA molecules, and protein isoforms. As a result, this study has contributed to improving the reference annotations of the human genome and transcriptome, with a focus on the annotation of features significant to the normal function of the heart. By re-analysing existing datasets, 166 putative genes were detected, 491 new non-coding and protein-coding isoforms were identified, 64 new potential protein isoforms or splicing protein by-product fragments were predicted, and 16 uncharacterised proteins were detected. In summary, we have identified strong transcriptomic candidates for verification by RT-PCR for Sanger sequencing, and proteomic isoforms for confirmation with mass spectrometry.

## Data Availability

Publicly available datasets were analyzed in this study. This data can be found here: Nanopore bulk cDNA sequencing data was accessed from the National Centre for Biotechnology Information (NCBI) database of Genotypes and Phenotypes (dbGaP) accession number phs000424.v9 (28–30). Illumina bulk cDNA sequencing data was accessed from the GTEx v8 project (30). CAGE peak data was accessed from refTSS4 (human 4.1). Homo sapiens (GRCh38.96) 3′-end sequencing data was accessed from the PolyASite atlas (v2.0).
