# Supplementary material for: Discovery and annotation of putative genes and novel RNA splice-isoforms in the human heart
Source: Front Cardiovasc Med. 2026 Sep 29;13:1920242. doi: 10.3389/fcvm.2026.1920242 (PMC13685430; doi:10.3389/fcvm.2026.1920242)
Supplement: Supplementary file 1 [file Datasheet1.docx]

Supplementary Material

# 1 Supplementary Data

# 2 Supplementary Figures and Tables

## 2.1 Supplementary Tables

**Supplementary Table 1.** Summary of novel RNA isoform characterisation and genomic features.

**Supplementary Table 2.** Cis-regulatory potential of putative lincRNA genes on neighboring protein-coding genes.

**Supplementary Table 3.** Gene ontology enrichment analysis for biological processes of hub genes identified in the turquoise module.

**Supplementary Table 4.** KEGG pathway enrichment analysis of hub genes identified in the turquoise module.

## 2.2 Supplementary Figures

Supplementary Figure S1. Analytical pipeline. The analytical pipeline encompasses several steps, starting with A. data acquisition and data pre-processing, which includes basecalling, quality checking and controlling, and two-pass SJ-informed read alignment. B. After this, transcripts are assembled and quantified with two different tools, Bambu and IsoQuant. Such transcriptional models are quality assessed, controlled and filtered using rule-based filters and the incorporation of additional transcriptomic data such as 3’end seq data and FANTOM5 cage peak data. C. The set of novel isoforms and genes that are structurally equivalent across both assembly tools are then utilised for further analysis. Further analyses such as differential gene expression, cis-regulatory potential of lincRNA genes, weighted gene co-expression network analysis, and sequence characterisation of protein-coding RNA isoforms are also performed.

**Supplementary Figure S2. Hierarchical clustering of unmerged module eigengenes by their dissimilarity.** Shown are module eigengenes (the expression profile of all genes within a module) identified in the dissimilarity topological overlap matrix (1-TOM) construction during weighted gene co-expression network analysis. Below the cut-height (red-line) indicates modules with a dissimilarity (y-axis) lower than 35%, or equally those with a correlation greater than 65% which are later merged.

**Supplementary Figure S3. Gene dendrogram with the original and merged modules.** Hierarchical clustering tree illustrating gene relationships based on the dissimilarity topological overlap matrix (1-TOM) on the y-axis, where lower branches indicate stronger co-expression. The original modules are the 41 modules identified by the dynamic tree cutting algorithm, and below are the 36 merged modules retained after merging highly correlated modules (PCC ≥ 0.65).

**Supplementary Figure S4. Correlation between modules identified from weighted gene co-expression network analysis and tissues.** Shown for each module (y-axis) and tissue (x-axis) are the Pearson correlation coefficients (PCC) and p-values (student asymptotic p-value) between the 29 modules and the atrial appendage, left ventricle, and skeletal muscle tissues.

Supplementary Figure S5. Hierarchical clustering of the atrial appendage, left ventricle, and skeletal muscle transcriptomes. Hierarchical clustering of the Bambu assembled transcriptomes of the atrial appendage (n=9), left ventricle (n=7), and skeletal muscle (n=9) is shown on the left y-axis and top x-axis. Both annotated and novel transcript models with variable expressions (SD > median SD) across the samples were retained for clustering. The correlation of transformed expression values (log2(CPM+1)) was calculated using Spearman’s correlation coefficient. The plots were generated with Bambu v.3.12.1 in an R v.4.51 environment. Abbreviations: HAA, heart atrial appendage; HLV, heart left ventricle; SKM, skeletal muscle; Sp.R, Spearman’s rho.

**Supplementary Figure S6. Karyotype plots representing top three upregulated and top three downregulated genes in left ventricle in comparison to atrial appendage.** For each represented human chromosome, the karyotype plot from top to bottom features the differential gene expression results, an ideogram of the chromosomes structure (bars), and a corresponding gene density plot (peaks). In the differential gene expression results panel, each gene is plotted according to its chromosomal location on the x-axis, and relative fold-change (log2FC) on the y-axis. The size of the point corresponds to the gene’s expression level. Genes whose expression is upregulated in the left ventricle is shown in red and those downregulated in blue. Points represent annotated genes whilst triangles represented novel genes. The top **three** upregulated and top **three** downregulated novel genes that were significantly (*p.adj* < 0.1) differentially expressed are labelled. Chromosomes containing such genes of interest were represented in the figure.

**Supplementary Figure S7.** The correlation between the tissues and the module eigengenes are illustrated in both the **A.** eigengene dendrogram and **B.** eigengene adjacency heatmap. **A.** Shown are the merged module eigengenes and tissue expression matrix by their distance [dissimilarity topological overlap matrix (1-TOM)] values (y-axis) where values closer to 0 indicate higher correlation. This was achieved by quantifying similarity (adjacency) by pairwise correlation between eigengenes and tissue trait data. **B.** The adjacency between the module eigengenes and tissues are illustrated in a heatmap.

**Supplementary Figure S8. Hub gene identification in the turquoise module.** Shown are the top 10% of most connected genes in the turquoise module in terms of their module membership (x-axis) and intramodular connectivity (y-axis).

**Supplementary Figure S9. Sequence characterisation summary of predicted ORFs.** Shown against the ORF sequence length of **A.** CodingTrx48, **B.** CodingTrx63, **C.** CodingTrx87, and **D.** CodingTrx79 are the results of TOPCONS topology prediction, InterProScan protein domain prediction, associated PANTHER gene ontology terms, *blastp* results, and NCBI’s conserved domain search results. The figure was generated with BioRender.

Supplementary Figure S10. TOPCONS topology prediction of proteins encoded by CodingTrx87 and CodingTrx79.

Shown at the top is the consensus TOPCONS topology prediction for the ORFs of **A.** CodingTrx87 and **B.** CodingTrx79, followed by the topology prediction results of five algorithms used to construct the consensus; OCTOPUS, Philius, PolyPhobius, SCAMPI, and SPOCTOPUS (left y-axis). Additionally, the homology to known 3D structures (TM protein TREK2 (PDB ID: 4bw5A) are shown, where gaps indicate sequence differences to the known structure. The predicted cellular locations (intracellular, transmembrane, and extracellular) are as indicated in the keys. The apparent free energy (ΔG_app)_ also known as the membrane insertion energy (ΔG) at each residue (bottom x-axis) is shown, where low values (right y-axis) indicate an energetically favoured membrane insertion.

Supplementary Figure S11. Membrane insertion energy graph of proteins encoded by CodingTrx87 and CodingTrx79.

Comparison of membrane insertion energies (x-axis) of **A.** CodingTrx87 and **B.** CodingTrx79 (black) to the insertion energies of a non-redundant set of soluble and transmembrane proteins. The number of known transmembrane protein structures (red) and soluble structures (blue) is indicated (y-axis).

Supplementary Figure S12. Raw short-read and long-read RNA sequencing data of the atrial appendage at the *KCNN3* gene.

Shown from top to bottom is the chromosomal location of the locus being visualised (indicated by a red bar), a panel including RefSeq annotated genes (shown in blue), and raw sequencing reads. An independent set of short-read RNA sequencing data (derived from the same tissue) is shown above the single replicate of atrial-appendage derived nanopore cDNA sequencing data. Visualisation of the reads was achieved with Integrative Genomics Viewer v.2.16.2.

Supplementary Figure S13. Raw short-read and long-read RNA sequencing data of the atrial appendage at the novel intergenic gene NovelGene23 and KCNJ3.

Shown from top to bottom is the chromosomal location of the locus being visualised (indicated by a red bar), a panel including RefSeq annotated genes (shown in blue), and raw sequencing reads. An independent set of short-read RNA sequencing data (derived from the same tissue) is shown above the single replicate of atrial-appendage derived nanopore cDNA sequencing data. Visualisation of the reads was achieved with Integrative Genomics Viewer v.2.16.2. Only a proportion of the short-read sequencing reads are shown.

Supplementary Figure S14. Raw short-read and long-read RNA sequencing data of the atrial appendage at *MAOB*.

Shown from top to bottom is the chromosomal location of the locus being visualised (indicated by a red bar), a panel including RefSeq annotated genes (shown in blue), and raw sequencing reads. An independent set of short-read RNA sequencing data (derived from the same tissue) is shown above the single replicate of atrial-appendage derived nanopore cDNA sequencing data. Visualisation of the reads was achieved with Integrative Genomics Viewer v.2.16.2.

Supplementary Figure S15. Raw short-read and long-read RNA sequencing data of the atrial appendage at *KCNK15*.

Shown from top to bottom is the chromosomal location of the locus being visualised (indicated by a red bar), a panel including RefSeq annotated genes (shown in blue), and raw sequencing reads. An independent set of short-read RNA sequencing data (derived from the same tissue) is shown above two replicates of atrial-appendage derived nanopore cDNA sequencing data. Visualisation of the reads was achieved with Integrative Genomics Viewer v.2.16.2.

Supplementary Figure S16. Raw short-read and long-read RNA sequencing data of skeletal muscle at *NADSYN1*.

Shown from top to bottom is the chromosomal location of the locus being visualised (indicated by a red bar), a panel including RefSeq annotated genes (shown in blue), and raw sequencing reads. An independent set of short-read RNA sequencing data (derived from the same tissue) is shown above the single replicate of skeletal-mucle derived nanopore cDNA sequencing data. Visualisation of the reads was achieved with Integrative Genomics Viewer v.2.16.2.
